# Intelligent Water Quality Assessment and Prediction System for Public Networks: A Comparative Analysis of ML Algorithms and Rule-Based Recommender Techniques

**DOI:** 10.3390/s26041392

**Published:** 2026-02-23

**Authors:** Camelia Paliuc, Paul Banu-Taran, Sebastian-Ioan Petruc, Razvan Bogdan, Mircea Popa

**Affiliations:** Department of Automation and Computing, Politehnica University Timisoara, 300006 Timisoara, Romania; camelia.paliuc@student.upt.ro (C.P.); paul.banu@cs.upt.ro (P.B.-T.); sebastian.petruc@upt.ro (S.-I.P.); razvan.bogdan@cs.upt.ro (R.B.)

**Keywords:** water quality assessment, machine learning, water drinkability prediction, decision tree algorithm, public water networks

## Abstract

An assessment and prediction system for the quality of public water networks was developed, using Timișoara, Romania, as a case study. This was implemented on a Google Firebase cloud storage system and comprised twelve ML algorithms applied to test samples for drinkability and used in predictions of upcoming samples. The system compares 17 water quality parameters to the World Health Organization and public reports of Timișoara drinking water standards for 804 samples. The system provides real-time data storage, drinkability prediction for the reservoir water system, and rule-based critical water recommendations for elementary treatment in samples. The most accurate and best-calibrated against random forest, gradient boosting, and Logistic Regression algorithms was the decision tree algorithm of the ML models. The experimental findings also determine the regions of the worst and best water quality and propose respective treatment. In contrast to previous research and structures, the paper demonstrates an approved stable solution for smart water monitoring, correlating practical deployment with sophisticated data-based conclusions. The results contribute to enhancing public health, enhancing water management measures, and upscaling the system for larger-scale applications.

## 1. Introduction

Water is needed to support a lifestyle, and the provision of clean and safe water is the need of the hour all around the globe. Even with the progress that has been achieved with water treatment and monitoring, over 1.1 billion people are not accessing safe water sources for the requirements of day-to-day living. This nagging issue has serious public health consequences, with an estimated diarrheal disease death rate ranging between 500,000 and 2 million annually due to the intake of unhealthy water, the World Health Organization (WHO) anticipates. In addition to the direct threat, even purportedly “safe” water is bound to cause chronic disease when its chemistry falls outside beneficial ranges [1,2].

In central cities, for instance, Timișoara in the European part of Romania, municipal water supplies are typically relied on for compliance. Nevertheless, discrepancies among water quality districts do occur due to changes in treatment effectiveness, pipe deployments, and environments. Unpredictable reporting and sampling at local water companies, for instance, AquaTim Timișoara, are often the method of surveillance. The reports, though comprehensive, are static and not so easily translatable to scaled-up, real-time prediction, evaluation, or useful recommendations [3,4,5,6].

Water quality is an overall outcome of the interplay among these parameters, being chemical (such as nitrates or pH), physical (such as conductivity or turbidity), and microbiological (such as *Coliform bacteria, E. coli*), situation-dependent, and nonlinearly dependent. The standard statistical analysis procedures do not account for these or provide prediction results. The procedures are also centralized and computing-oriented, rather than real-time [4,7,8,9,10,11,12].

As the use of sensor networks and remote monitoring increases, the cost of installation can be high, and these systems may only be used to monitor specific areas within the city’s water supply. While it is possible to retrieve the data, there is still a significant gap in the information available to decision-makers in the water management and health departments, as the solutions available to us today do not offer the use of integrated prediction and measurement, only widespread monitoring and treatment recommendations. As such, smart water monitoring systems based on wireless sensor network technology have been extensively proposed to enable the continuous and automatic measurement of water quality in the water distribution network. However, such systems heavily rely on the deployment of a large number of wireless sensors in the water network, which is expensive to install and maintain. This results in a sparse coverage of the water network and protection of the water quality in localized points of the water network [13]. Centralized in-network water monitoring systems have mainly focused on addressing the needs of data acquisition and anomaly detection in water infrastructure. However, such systems have largely failed to effectively translate the acquired data into consumable information for non-expert stakeholders and water consumers [11]. More recently, water monitoring systems based on the IoT and virtual sensing technologies have been proposed to enable the prediction of water quality based on limited physical measurements. However, such systems have largely focused on the estimation of water quality parameters and treatment recommendations, rather than the end-to-end prediction of water quality [10]. Water monitoring systems based on edge and fog computing technologies have been recently proposed to enhance the transmission of data in the water network. However, such systems are still hardware-intensive and mostly focused on the implementation of specific water monitoring objectives without the end-to-end prediction of water quality [14]. Even the recently proposed water quality forecasting systems, which are mostly near real-time in nature, have been largely research-oriented and centralized in their implementation. Moreover, such systems have been difficult to adapt to the implementation of water management and public health decision systems in the city [15].

In order to ensure that we have safe drinking water, we should develop an approach that adapts, uses smart insights, and is budget-friendly. The approach should be able to continually monitor water quality, provide reliable potability estimates, and offer practical recommendations that meet global safety standards. It should also incorporate the newest data, as well as historical data, and utilize the latest in machine learning while providing useful recommendations to the end user.

The motivation of the presented project originates from the need to increase awareness regarding the challenges inherent to the field measurement and maintenance of the quality of drinking water, particularly in under-resourced or patchily observed locations. For both rural and urban settings, an extensive lack of accessible real-time knowledge and affordable water test apparatuses hinders the early detection of contamination and the implementation of effective treatment strategies. Such issues beget the overall imperative for smart, accessible, adaptive systems that are capable of water quality measurement and predictive support for decisions at the local and wider public health levels.

Timișoara, a European city, was chosen for the case study due to the fact that public water reports are easily accessible and the diversity of the areas that were to be administered, offering the ideal environment for piloting a scalable solution. The fact that even cities that possess an integrated water system lack a centralized and smart system for water monitoring and analysis underlines the importance of the work at hand.

Through the integration of cloud technologies, machine learning, and rule-based algorithms, this project adds a working prototype capable of assessing and forecasting water drinkability. Its successful pilot in Timișoara also sets an example that is easily transferable to other cities worldwide. Section 2 lays out an extensive literature review, featuring the latest developments in water quality observation, machine learning usage, and recommender system integration. Section 3 outlines the envisioned smart system with intricate detail, comprising architecture, a pipeline for processing the data, algorithmic aspects, and the runtime environment. Furthermore, it describes the experimentation strategy and the metrics for evaluation that are utilized for the analysis of system performance while implementing several machine learning models. Section 4 presents the major findings, comprising accuracy thresholds, water quality interpretation for each city region, and recommendations for action. Section 5 provides an extensive discussion of the realization, limitations, and scalability potential to other cities. Lastly, Section 6 summarizes the findings and maps the future research and real-world implementation.

## 2. Literature Review

The supply of convenient and clean drinking water has become one of the main challenges of our era. Under the growing pressure of climate change, urbanization, and industrialization, water quality has declined, encouraging scientific research focused on water monitoring, treatment, and predictive systems. One of the main studied topics of this type of research is the incorporation of artificial intelligence (AI) and machine learning (ML) techniques into water management models, providing powerful tools to work with large and complex data sets for the derivation of predictive insights [10,13,16,17,18,19].

### 2.1. AI and ML in Water Quality Prediction

Due to their capacity to capture the multivariate and non-linear relationships present in environmental datasets, artificial intelligence and machine learning techniques are increasingly being used for water quality modeling and classification, according to recent studies [20]. Several water quality datasets have been used to assess a variety of supervised learning algorithms, such as decision trees, Support Vector Machines, Artificial Neural Networks, k-Nearest Neighbors, and ensemble-based techniques [21,22,23,24,25].

Although some studies have achieved good results with decision tree models, particularly in a regulated environment for classification problems [21,24,25], other studies have obtained better results with Support Vector Machines, neural networks, and ensemble methods like random forest, LightGBM, and stacking classifiers [22,23,26,27,28]. The above results demonstrate that features of the dataset, like the number of samples, feature distributions, and the presence of missing values, have a significant effect on the performance of the models.

In general, the majority of the earlier studies have focused on selecting a best-performing classifier for a particular set of data, with a minimal emphasis on cross-algorithm evaluation with unified preprocessing and validation. This, again, highlights the importance of using prediction models in conjunction with decision support tools, as our study indicates. There is also a need to develop a broad, standardized framework to test machine learning models under similar experimental conditions. The current study has also focused on providing recommendations with the help of a rule-based approach, which is not seen in earlier studies.

### 2.2. Comparative Studies of ML Algorithms

Comparative studies on various machine learning models for water quality classification and Water Quality Index (WQI) prediction have increasingly been a topic of interest in various studies [1,29,30,31,32]. These studies have compared various machine learning models, such as linear, tree-based, neural, and hybrid models, for water quality classification, drinking water classification, irrigation suitability assessment, and WQI prediction.

Overall, the studies have confirmed that no particular model has been superior to others for various water quality classification and prediction tasks. The neural and hybrid models have been the best options for continuous WQI prediction, whereas tree-based models, such as decision tree and random forest, have been observed to perform well for binary classification-based regulatory scenarios.

To ensure the effectiveness of various machine learning models for various water quality classification and prediction tasks, this study conducted a unified experimental protocol to compare various machine learning models, as discussed earlier.

### 2.3. Integration of IoT and ML for Water Analysis

In addition to predictive modeling, a number of research papers have also explored the integration of machine learning with Internet of Things (IoT) technologies for the purposes of automated alerting, anomaly detection, and real-time water quality monitoring [33,34,35,36]. To ensure the continued surveillance and monitoring of water distribution systems, the proposed systems integrate multi-sensor technologies with machine learning-based classifiers.

On the contrary, the proposed machine learning-based methods have also been explored for the detection of particular microbial hazards and the facilitation of warning systems, such as the detection of *Escherichia coli* and the generation of threshold-based alerts [37,38]. Earlier research has focused on the benefits and possible applications of using monitoring and alerting systems, which are powered by machine learning. Most of these studies have focused on real-time sensing and detection. However, this study will be different in that it will be comparing the models used in predicting the class of water potability.

### 2.4. Water Treatment Approaches

The earlier works have greatly focused on water treatment modeling and optimization. They include studies on turbidity and aluminum control, the efficiency of the coagulation process, and bacterial reduction measures such as induced bank filtration [39,40,41]. These works, among others, attest to the significance of process and level modeling in enhancing treatment effectiveness and operational efficiency.

On the other hand, some of the studies have been directed towards decision support systems and sensor-driven monitoring platforms for assessing finished water quality and for contaminant detection [42,43]. In addition to that, regional-scale modeling studies have been conducted to understand chemical reactions and the optimization of purification plants in order to achieve higher scalability and cost efficiency [44].

Nonetheless, the majority of the current solutions focus on reactive control and post hoc assessment. They pay little attention to proactive, data-driven recommendation mechanisms that integrate multiple nonconformities to suggest targeted interventions.

This is the reason why the present work proposes the development of smart recommendation components that will serve as a complement to predictive analytics.

### 2.5. Water Recommendation Systems

Less time has been spent addressing water suggestions, especially for public networks. Mahmoud et al. [45] designed a rough mereology-based recommender to predict pH pollution from the dataset provided by the EPA with a 0.34 mean absolute error.

In a different application area, Rahim et al. [46] built an LSTM-based system to individualize consumer behavior for sustainable water use. However, for conservation purposes, water service personalization is hinted at.

This ongoing assignment integrates basic but effective rule-based algorithms to recommend:

- Water with the maximum and minimum acceptable dissolved solids.
- Samples with the lowest levels of water hardness.
- Treatability for non-conforming samples.

As the author recognizes, this integration of smart classification and simple recommendations has not been practiced yet with the system of municipal water quality.

### 2.6. Water Distribution System Engineering

In recent studies of water distribution system engineering, the effectiveness of water quality assessment was shown to be strongly dependent on the different monitoring strategies implemented and the placement of water quality sensors within a network. The work of Piazza et al. [47] discusses the impact of hydraulic conditions on the distribution of contaminants in water distribution networks. It highlights the significance of flow dynamics. At the same time, it is noted that transport processes such as diffusion and dispersion affect the performance of detection methods.

### 2.7. Gaps and Limitations of the Cutting-Edge Solutions

The following key limitations are common:

- End-to-end systems are not offered: most classify or forecast, but none do all at once under the same system.
- Limited practical use: Most are theoretical or simulated. Not very many are exercised with full-scale publicly accessible datasets or with real-world use.
- Ignoring localized variability: Most models do not consider spatial heterogeneity for the water distribution system. Intercity variations are not studied at all.
- Failure to compare algorithms for the same dataset: very few studies cross-validate a number of ML models on the very same water datasets, thus not enabling actual performance comparison.

## 3. Methods and Solutions

The gaps found in the current systems and literature are directly targeted in this research endeavor through the conceptualization and development of the Intelligent Water Quality Assessment and Prediction System. Our solution, exemplified in the city of Timișoara, is all-encompassing, practical, and scalable. It integrates real-time cloud storage, rule-based analytics, and sophisticated machine learning models.

We propose an intelligent water quality assessment and prediction system for urban public networks. The main objective of this paper is to evaluate the drinkability of water samples based on established health standards, utilizing machine learning (ML) algorithms for prediction and rule-based logic for recommendations. Our research follows the following steps: the acquisition and preprocessing of public water quality data from municipal reports; feature selection and data labeling according to World Health Organization (WHO) potability thresholds; the implementation and comparative evaluation of twelve supervised ML algorithms for binary classification (drinkable and non-drinkable); the development of an engine of recommendation for suggesting the optimal water samples and possible treatments; and, finally, system deployment implementing a cloud-based database for real-time accessibility and scalability. For the data sampling, we opted for a real-time cloud database that was previously integrated with Google Firebase. The database stores water samples as JSON objects.

We divided the utilized dataset into training and testing subsets using a proportion of 80/20, respectively. The split was performed randomly, without the use of stratification and without enforcing any fixed random seed. Furthermore, cross-validation and repeated sampling were not applied; all reported performance metrics, therefore, correspond to a single train/test split. While this setup may lead to optimistic estimations of performance, particularly for the tree-based models, it also enables efficient exploratory comparisons between different classifiers.

In the present study, we constructed the labels indicating the potability of water based on compliance with the World Health Organization (WHO) guideline thresholds for each physicochemical parameter. Each sample was labeled as either non-potable (if at least one measured parameter exceeded its corresponding regulatory limit) or potable (if the measured parameters did not exceed the corresponding regulatory limit). As a result, the classification task is focused on the aspect of regulatory compliance, not the intrinsic or toxicological definition of potability. The labels are based on threshold-based criteria, not independent measures of the intrinsic safety of the water.

In the binary classification task we chose, we treated the potable class as the positive class for the computation of ROC and precision–recall metrics. The non-potable class represents regulatory non-compliance, and it is also evaluated accordingly through complementary class-wise metrics.

The interpretation of the ROC AUC and the precision–recall AUC must be considered from the perspectives of class distribution and of the definition of the positive class. With the potable class being treated as the positive class and representing the majority of samples, the ROC AUC values could appear with a value of around 0.5 even when the classifier performs well on the minority non-potable class. In contrast, the precision–recall curves remain more sensitive to class imbalance, providing a clearer indication of performance when the focus is on correctly identifying minority or critical cases.

### 3.1. Proposed System

**Figure 1:**
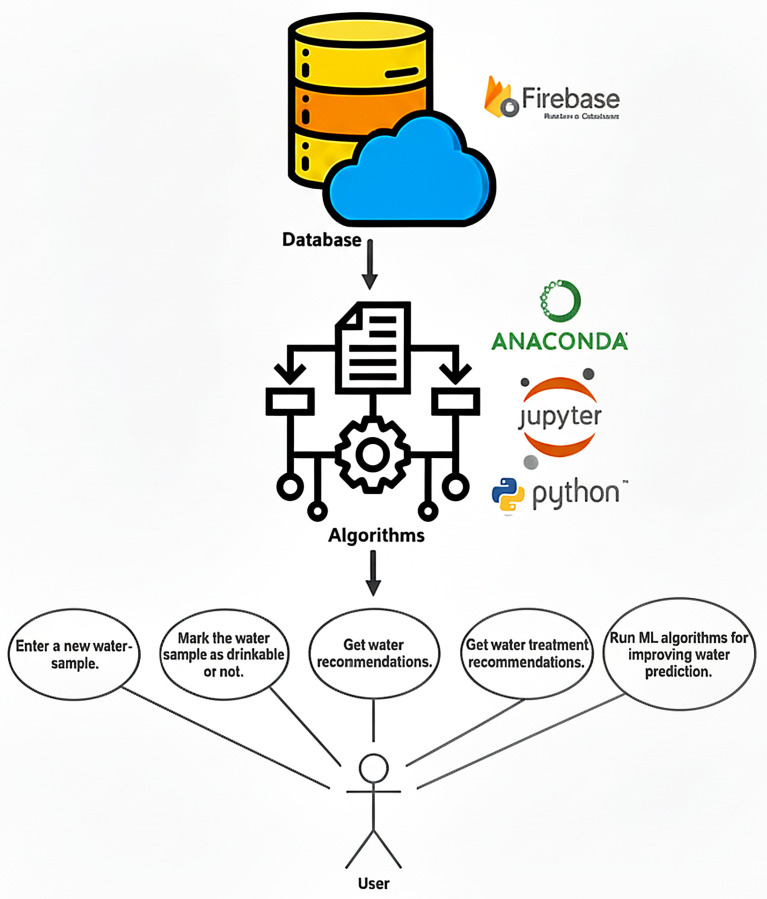
Design of the proposed system.

**Figure 2:**
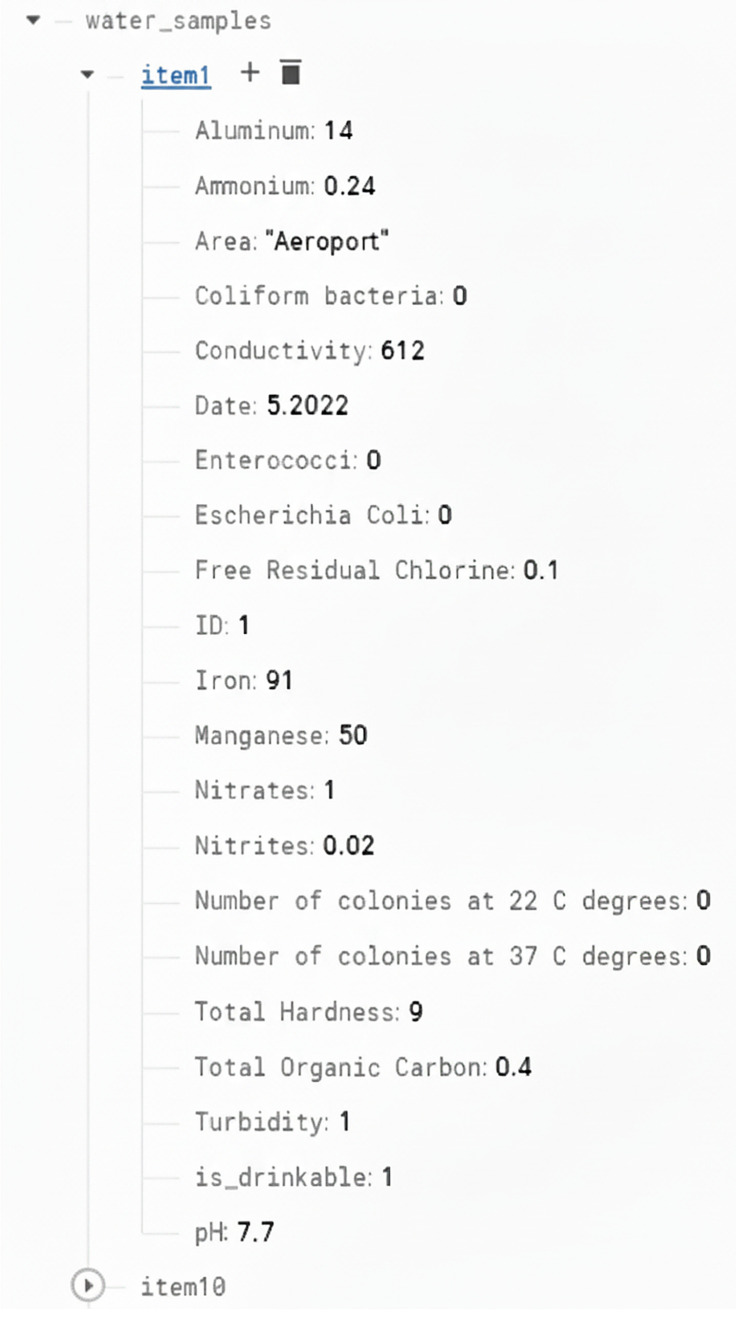
An entry extracted from Firebase.

The entire system’s architecture can be observed below in Figure 1:

- Data storage: a real-time cloud database implemented using Google Firebase stores water samples as JSON objects, offering secure, accessible, and live-updatable storage.
- Data acquisition and processing: A total of 804 public PDF water-quality reports were extracted from the official AquaTim S.A. public web portal [48] and converted into a structured dataset containing 17 parameters per sample. An example of a sample can be observed in Figure 2. Data parsing, data enrichment, and analysis were then applied using Python 3.8 in Jupyter Notebooks 6.x, together with libraries such as, for example, Seaborn 0.11.x and Plotly 5.x for visualization and statistical insights. These processes’ output was a dataset that we then used with the ML module, including twelve different classification algorithms ranging from simple linear models to more complex methods, such as ensemble and tree-based methods.
- Recommendation engine: rule-based logic provides recommendations for best water, water with extreme pH values, and treatment suggestions for samples outside acceptable thresholds.

### 3.2. Addressing Gaps in Real-Time Integration and Implementation

Whereas the majority of the methods that have been proposed so far remain mostly at the theoretical or simulated implementation level [49], the system we propose is fully operational and designed to work with actual water quality data, manually gathered from several subregions of the Timișoara area. This is important, as the actual validation of the results is often missing from the existing literature. All the water samples from the database are comprised of a time stamp and a location, enabling trend tracking, anomaly detection, and inter-zone comparisons.

Furthermore, the use of Firebase ensures that the system is not committed to a static dataset, but it can always be updated, and it is possible to incorporate new samples manually or, for future deployments, automatically through scraping or IoT connectivity.

### 3.3. General Algorithm Evaluation to Benchmark

**Figure 3:**
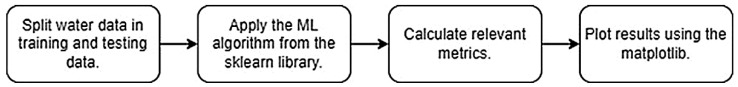
Function flow for ML algorithms.

In this paper, we compare twelve supervised machine learning models by applying them to the same common real-world water quality dataset. While most of the literature is concerned with a smaller selection of models, this paper proposes a comprehensive benchmark over a broader range, presenting an overview of the corresponding performance, interpretability, and suitability concerning water potability analysis and predictions. Therefore, for Figure 3, the flowchart for the water prediction system is shown.

The ML models that are called upon here are as follows.

- Linear regression and logistic regression: These are basic models that are commonly employed as baselines for classification tasks. Linear regression, though mostly not applied for regression tasks, can accentuate relationships among features. Logistic regression is more suitable for binary classification and uses weight coefficients that are interpretable, showing each feature’s contribution to the probability of a class. The utilized linear regression algorithm models a relationship between an outcome variable, denoted as Y, and a set of independent or input variables, denoted as X, under the assumption of a linear relationship between variables [50]. This method produces a best-fit line used to minimize deviations between predicted and actual values. Logistic regression is a type of statistical modeling that calculates the probability of a binary event, such as drinkable or non-drinkable water, based on a single or multiple independent variables [51]. This contrasts with linear regression, which calculates continuous outcomes, as logistic regression is created for binary classification problems only.
- Naïve Bayes: It is a probabilistic classifier that uses Bayes’s theorem under the assumption that the attributes are independent. It is fast computationally and appropriate for high-dimensionality datasets; however, the naïve assumption decreases the accuracy if the attributes are highly correlated. The naïve Bayes classifier is an effective classification algorithm using Bayes’s theorem, which remains valid, assuming the independence between features, given the class label [52]. Its role as a practical algorithm lies in its speed, ease of implementation, and capability of classifying high-dimensional data.
- Decision tree: A tree model that splits the dataset hierarchically, recursively, with the help of the feature thresholds, to form a decision hierarchy, it is very interpretive and is applicable to rule-based areas like environmental monitoring, but it suffers from overfitting for noisy datasets [53,54,55,56]. The decision tree algorithm is a supervised learning algorithm that may be suitable for both classification and regression problems. It behaves as a flowchart, in which an internal node will represent a feature, a branch will represent a decision rule based on a feature, and a leaf node will represent an outcome or a prediction. It tries to carve out subsets from the dataset, which are more homogeneous with respect to a target variable, through repeated partitioning over feature values. At every decision point, the split that separates out classes best or that improves predictiveness best is chosen by the algorithm [53,54,57,58].
- Random forest: A set of decision trees built from randomly sampled features and instances, it diminishes the overfitting and variance issues inherent to individual trees, with the advantage of reporting feature importance scores. Random forests are ensembles that learn, which are comprised of a collection of decision trees, with each tree being built with a randomly drawn subset of data, as well as input features [59]. Diversity at each split with a random vector helps minimize overfitting as far as possible and generalizability, as each tree is built with a different set of features.
- Support Vector Machine (SVM): powerful classifiers that construct an optimal hyperplane for class separation in a very high-dimensional space, SVMs are robust for highly complicated or sparse datasets but computationally costly and highly sensitive to parameter tuning [55,60,61,62].
- K-Nearest Neighbors (KNN): A non-parametric method that classifies the majority vote among the nearest neighbors within the space of the features, KNN is easy to implement and simple to program, but it is susceptible to too great a computational cost and sensitive to irrelevant or scaled attributes. K-Nearest Neighbors is a supervised learning algorithm applicable to classification, as well as regression problems. It works on a similarity basis, estimating the output for any input by considering the mean value of its *k* nearest neighbors in the training set. Its neighbors are identified predominantly through a distance function measuring the closeness in the feature space [55,58,63,64].
- AdaBoost: It is an iterative process that aggregates weak learners iteratively by going through the process of updating the misclassified samples’ weights so that the model focuses on the problematic instances. It is highly effective for improving accuracy, especially for datasets that are imbalanced. AdaBoost, which stands for “Adaptive Boosting,” is the most famous boosting algorithm that falls under the ensemble family of methods looking to enhance predictive performance by forming a combination of several weak learners [65]. AdaBoost stands out for its ease of implementation and vast selection of base classifiers, frequently producing powerful results with a minimum amount of parameter tuning. The methodology utilizes the greedy paradigm, adding weak classifiers in a sequential manner and having different weights adjusted to improve the model by gradually developing a stronger classifier.
- Gradient boosting: A sophisticated boosting method through which the next learners are educated to rectify the mistakes of the current ensemble using gradient descent optimization. It is highly predictive but involves a higher computational expense and lower overall interpretability. Gradient boosting is a form of ensemble learning that builds a powerful prediction model by iteratively combining weak learners. We train each new model in order to reduce a given loss function, for instance, the Mean Squared Error for regression or cross-entropy loss for classification. This causes the model, with each subsequent iteration, to decrease the overall prediction error [55,66,67,68].
- Bagging Classifier: A methodology of bootstrap aggregating capable of creating many base estimators that are trained on randomly sampled sets of the data and combine the predictions precisely to minimize variance, it is useful for stabilizing models and working with overfit-prone learners such as decision trees. The Bagging Classifier uses an ensemble method that produces multiple instances of a base predictor by making bootstrap samples of the training set and pooling their outputs [69]. For regression, outputs are averaged, whereas for classification, a majority vote selects the output.
- Linear Discriminant Analysis (LDA): A statistical technique that maps the data to a lower-dimension space to achieve class separability maximization, it is assumed to be normally distributed with equal covariance among classes, so it is fine for linearly separable data. Linear Discriminant Analysis (LDA) is a classification method that seeks to project features in a feature space in a lower dimension by optimizing between-class variance to the within-class variance ratio, improving class discriminability [70].
- Neural Networks (Multilayer Perceptron): The model included a neural network consisting of a fully connected (dense) layer. The built MLP is suited for well-structured tabular datasets and can capture intricate, nonlinear associations between attributes. Although the model obtained competitively accurate results, it is not very interpretative relative to lighter-weight classifiers and needs to be laboriously hyperparameter-tuned. A Multilayer Perceptron or MLP is a family of feedforward artificial neural networks that consist of a single or several hidden layers, an output layer, and an input layer. The different layers contain several artificial neurons that are fully interconnected with neurons in adjacent layers. MLP models can represent complex, non-linear relations with the use of an activation function such as ReLU or a sigmoid function. During training, the network adjusts its connection weights with a backpropagation algorithm supported by a gradient descent for loss function minimization. MLP models are faster for classification tasks with structured, tabular data, including instances where feature interactions are not necessarily linear [55,71,72,73].

Each model was evaluated with multiple assessment metrics, such as accuracy, the true positive rate, the false positive rate, precision, and ROC-AUC, in order to yield a comprehensive assessment of predictive accuracy for the binary class task (drinking versus non-drinking water). Accuracy indicates the overall percentage of samples classified correctly, providing a rough overall picture of performance, but it is potentially deceptive for class-imbalanced datasets. The true positive rate (or recall or sensitivity) gauges the model’s reliability in correctly classifying non-drinking water samples, the chief concern for system deployment in public health applications. Conversely, the false positive rate describes how frequently the model mislabels drinking water samples as not potable, compromising public confidence in system advice. Precision estimates the percentage of samples that the model rightly predicts to not be drinkable, facilitating an assessment of the model’s credibility for the possibility of issuing alerts for drinking water contamination. Lastly, the ROC plot graphs the balance between true and false positive rates at different thresholds, with the Area Under the Curve (AUC) encapsulating the model’s aggregate power for discrimination and distinguishing positive from negative samples. Collectively, these metrics provide threshold-dependent and threshold-independent perspectives on model behavior.

The test results for the decision tree model were 100% accuracy and perfect calibration, validating other researchers’ findings [21,24,25], but they were now applied to an actual municipal dataset. The next best performers were gradient boosting and random forest, but with rather poor reliability for calibration. Through this extent of comparison, the system enables decision-makers to choose the best-suited model, considering various trade-offs (such as accuracy versus explainability).

### 3.4. Intelligent Recommendations Based on WHO Guidelines

In addition to ML prediction, the system also provides actionable, easy-to-use recommendations in accordance with the standards of the WHO [74]. The following are incorporated:

- Water with the highest/lowest pH: ranked and visualized per neighborhood, convenient for consumers with health concerns (such as kidney disease).
- Best water: based on a rule-based score, all water samples receive a rank by closeness to optimal WHO values for all parameters (for example, TOC, chlorine, and coliforms).
- Treatment suggestions: basic but meaningful treatment recommendations (such as UV disinfection, reverse osmosis, or activated carbon filtration) are suggested based on detected anomalies.

This is a novel functionality amongst the presented list of work. The vast majority of systems employ detection or modeling [20,33], but very few offer next-step recommendations that are sample-characteristic.

### 3.5. Case Study Deployment in Timișoara

The Timișoara municipality provided an ideal pilot test situation because of the following elements:

- The evaluation of open water quality reports from AquaTim.
- Variation and frequencies for neighborhood attributes.
- Variability occurring in real-world parameters like TOC, the presence of bacteria, and pH.

### 3.6. Scalability and Future Direction

One of the advantages of this system is extensibility. As it is both cloud-based and a modular system,

- It is easy to add new ML models.
- It is possible to integrate real-time sources of data (such as IoT sensors).
- Mobile dashboards could be built for wider accessibility.
- Reporting public APIs for community health could be designed.

In addition, the system architecture is extensible for other cities. The system can be installed and run directly for any other city with a frequent reporting of water or readily accessible sensor observations.

### 3.7. Study Area

**Figure 4:**
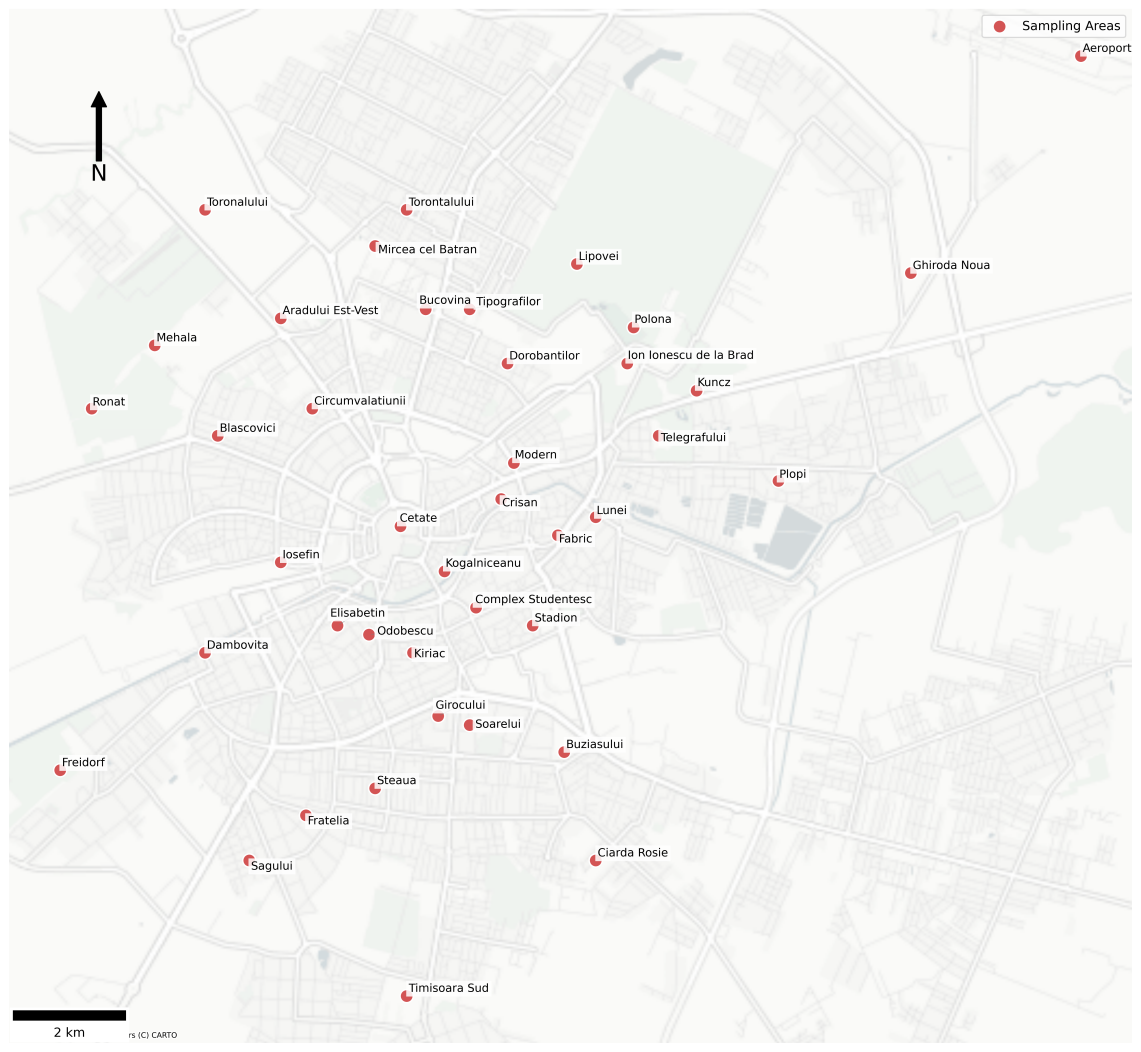
Map indicating the sampling locations in the city of Timișoara.

In this paper, we chose a study area that included measurements collected from a multitude of monitoring locations distributed across the entirety of Timișoara, the selected city in the western part of Romania, covering residential, commercial, and mixed-use districts. Namely, these areas include districts such as Cetate, Circumvalațiunii, Iosefin, Fabric, Dâmbovița, Girocului, Soarelui, Lipovei, Mehala, Freidorf, Buziașului, Torontalului, and Timișoara Sud, as well as peripheral zones such as Aeroport and Aradului Est–Vest. The spatial diversity present in the monitoring sites allows the dataset to capture a vast variability in scenarios across the urban water distribution network. This study focuses on data originating from a broad selection of urban districts of the city of Timișoara, all directly representative of the city-wide drinking water supply system. Geographically, the areas are similar due to the relative lack of geological diversity in the territories of the city of Timișoara, being mainly fields without mountainous areas. A map containing the sampling locations in the city of Timișoara is presented in Figure 4.

### 3.8. Ethical, Public Health, and Policy Implications

By translating complex ML results to clear directions, this system also fills the broader societal need for clear and interpretable health information. Citizens are able to make informed decisions about the water that they drink, and political leaders are able to invest in the proper treatment with data-informed priorities. Moreover, using public datasets ensures that the system adheres to the principles of open science and civic tech, enabling other researchers to test, critique, and improve the system with increased functionality.

## 4. Results

In this section, we present the empirical results of the water quality evaluation system through the application of twelve machine learning (ML) models to a dataset comprising 804 water samples. Each model is assessed with an identical set of performance metrics, such as accuracy, precision, recall, ROC-AUC, and calibration, in order to classify performance for drinkable versus non-drinkable water. Beyond the algorithmic comparison, the present section also reveals an analysis of potability distribution among diverse neighborhoods for the city of Timișoara. It then determines the prevalent causes for non-compliance with international norms and displays the output for the integrated recommendation module. These results support the predictive power of the system and serve to illustrate the practical value for the direction of water safety actions and for water-related public health decision-making.

Using the proposed system, the following findings were derived:

- 80.2% were drinkable, and 19.8% were not satisfactory to achieve the standard for the WHO.
- The Airport Area exhibited the highest average pH (7.9), with Telegrafului exhibiting the lowest.
- The Kiriac Area achieved the best overall water quality that matches the WHO standards.
- Girocului and Complex Studențesc needed the highest frequency intervention, for the most part, for TOC and bacterial load.

These products were made possible due to the modularity of the system, so that any stakeholder (researcher, operator, or citizen) could integrate data, run predictions, and analyze trends.

### 4.1. Linear Regression

**Figure 5:**
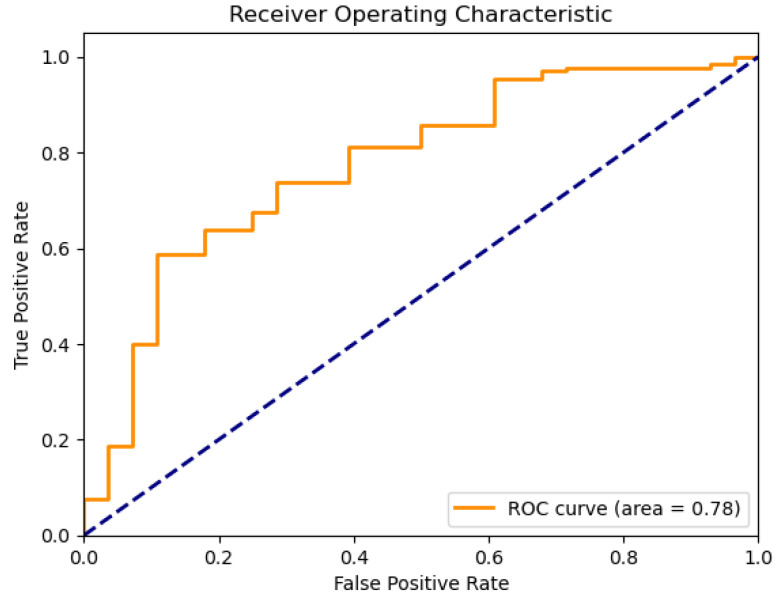
Linear regression: ROC curve.

**Figure 6:**
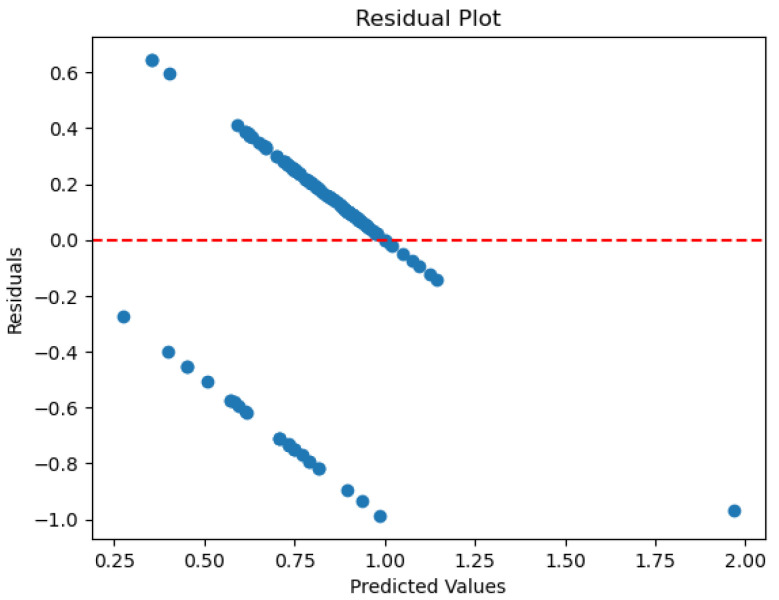
Linear regression: residual plot.

In applications using the existing water quality dataset, the utilized linear regression model exhibits relatively good classification accuracy. Its ROC curve (Figure 5) indicates an AUC of 0.78 against the dashed line indicating random guess, implying a good discriminatory ability between drinkable and non-drinkable samples. Further, its residual plot (Figure 6) exhibits overestimation, as well as underestimation patterns, implying that this model fails to consistently account for the underlying distribution of variance in the data. In this application of linear regression to predict water drinkability, the accuracy of the model is evaluated with typical regression measures, such as the mean square error (MSE) and the coefficient of determination ($R^{2}$). For this dataset, the model produces an MSE value of 0.13, with an $R^{2}$ score of 0.11. The MSE value indicates that, on average, the squared discrepancy between predicted outcomes and true outcomes was 0.13 units, which is a larger value—meaning that, overall, accuracy was poor. The implication from the $R^{2}$ score, meanwhile, that just 11% of the variation in drinkability outcome was explained by this model, coupled with a generally poor quality of predictions, suggests that overall, this model fits poorly. What this indicates, more generally, is that linear regression is just not suitable for this binary outcome classification problem—a result that was expected, nonetheless, since the technique is, by nature, suited for outputs that are continuous, not categories.

### 4.2. Logistic Regression

**Figure 7:**
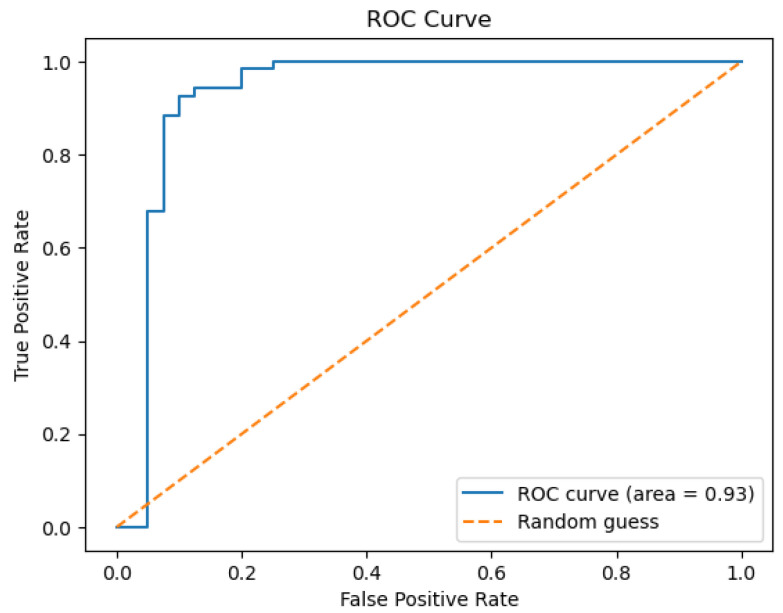
Logistic regression: ROC curve.

**Figure 8:**
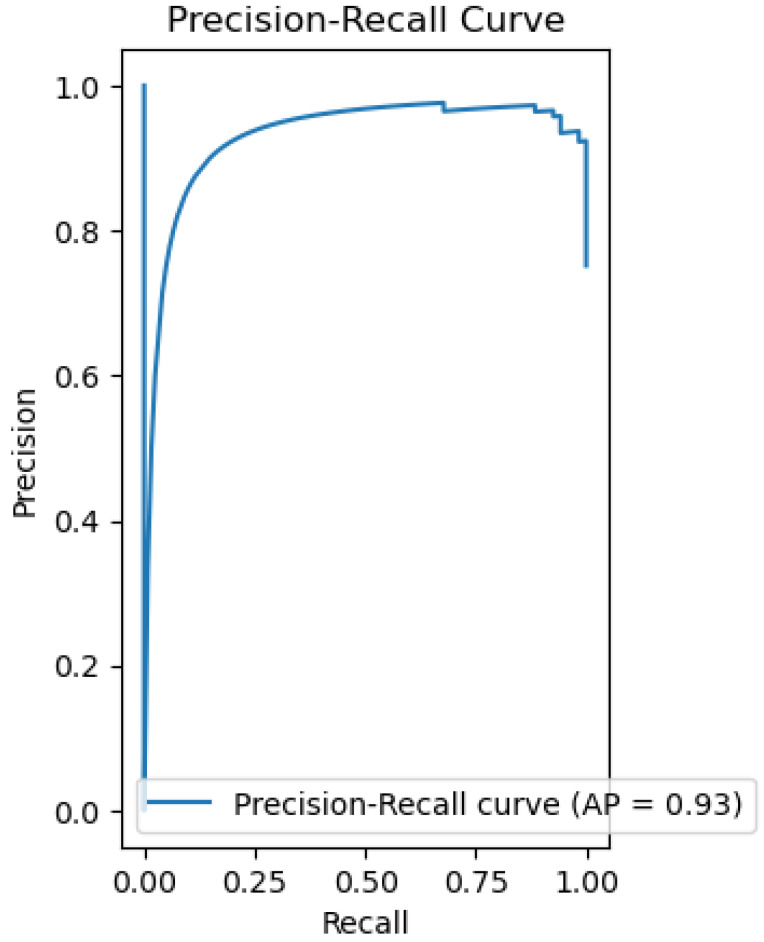
Logistic regression: precision–recall curve.

**Figure 9:**
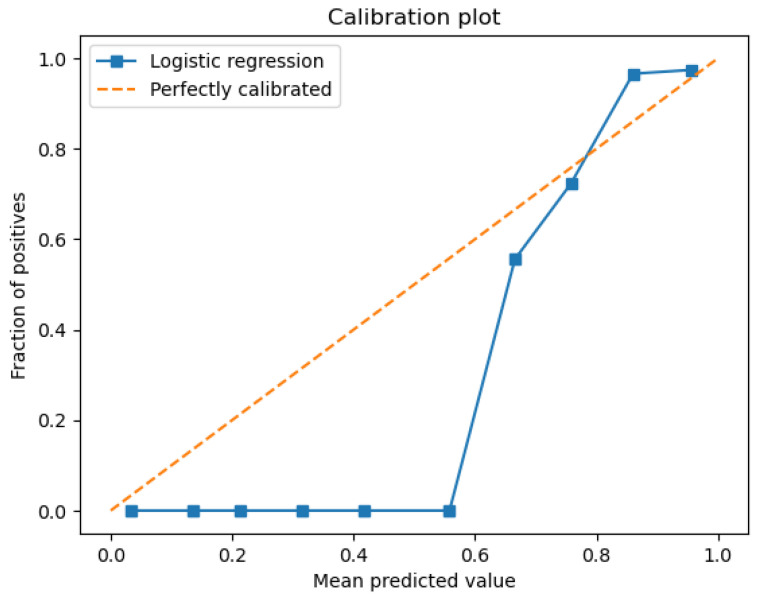
Logistic regression: calibration plot.

**Figure 10:**
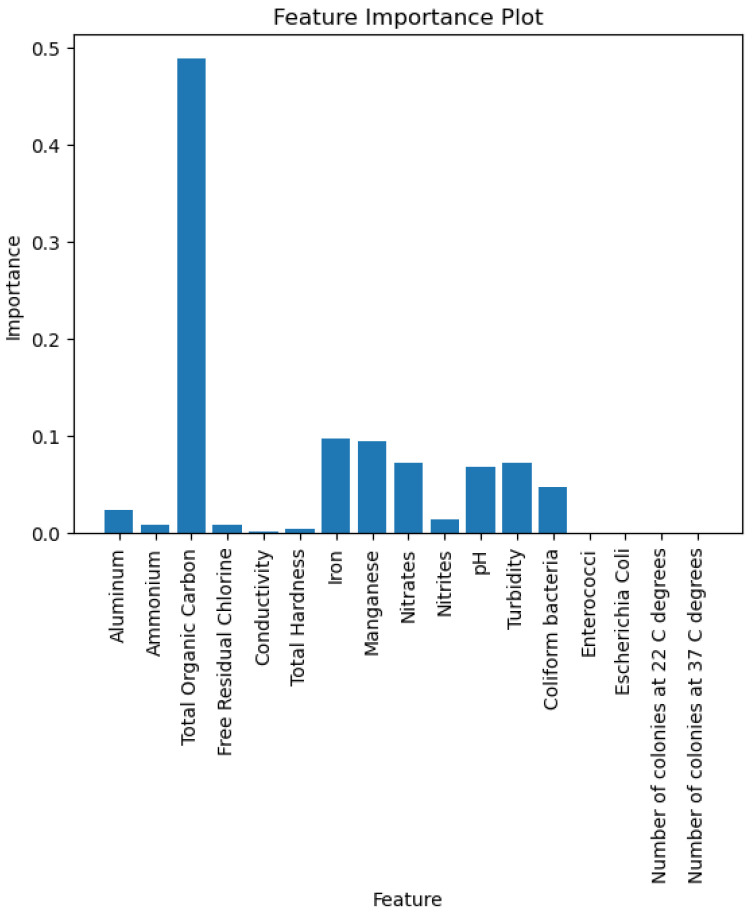
Logistic regression: feature importance.

The model is able to provide good discrimination between the drinkable and non-drinkable samples. The ROC curve (Figure 7) shows that the AUC is 0.93, implying that the logistic regression is giving higher probabilities to the right class. The precision-recall curve (Figure 8) shows high precision and recall values, implying that the model is keeping the false positive and negative values low. The calibration plot (Figure 9) shows that the predicted values match the actual values, implying that the predicted values are not too high or too low. The feature importance plot (Figure 10) shows the parameters used in the classification, giving the model more interpretability by showing the parameters used in the classification of the water quality indicators. The accuracy of the model is 86.3%.

As shown in Figure 10, total organic carbon stands out as having the greatest impact in the importance distribution, followed by iron and manganese at a moderate level, while pH, Turbidity, Nitrates, and *Coliform bacteria* have lower impacts, all below 0.1. Even though these parameters have lower individual impacts, they are still important in the drinkability analysis because they represent different aspects of water quality.

### 4.3. Naïve Bayes

**Figure 11:**
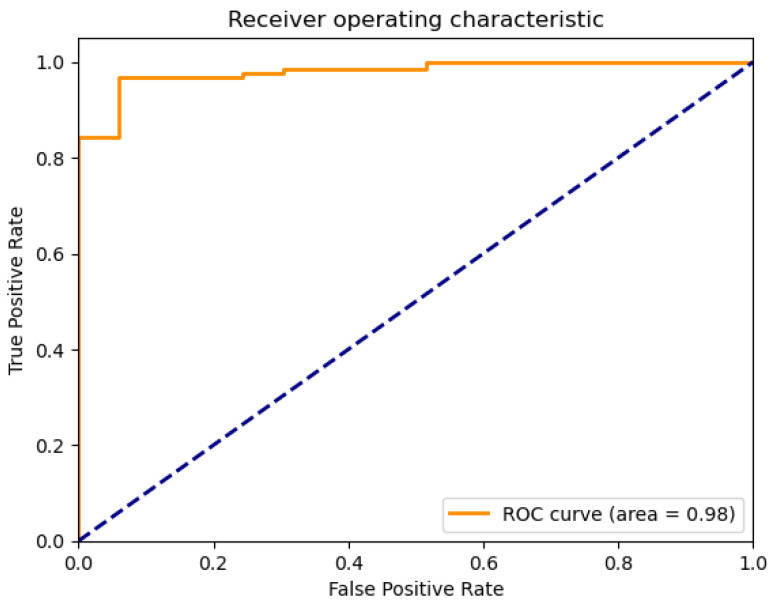
Naïve Bayes: ROC curve, where the dashed line is representing Random guess.

**Figure 12:**
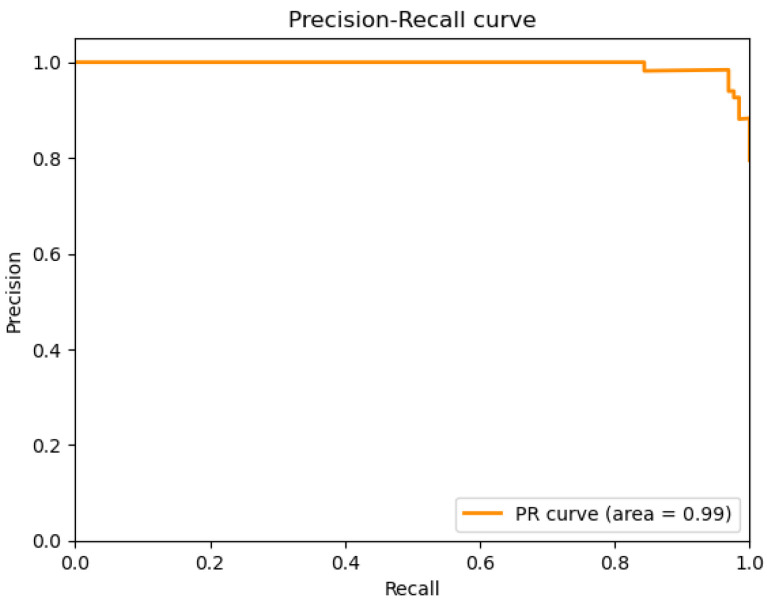
Naïve Bayes: precision–recall curve.

**Figure 13:**
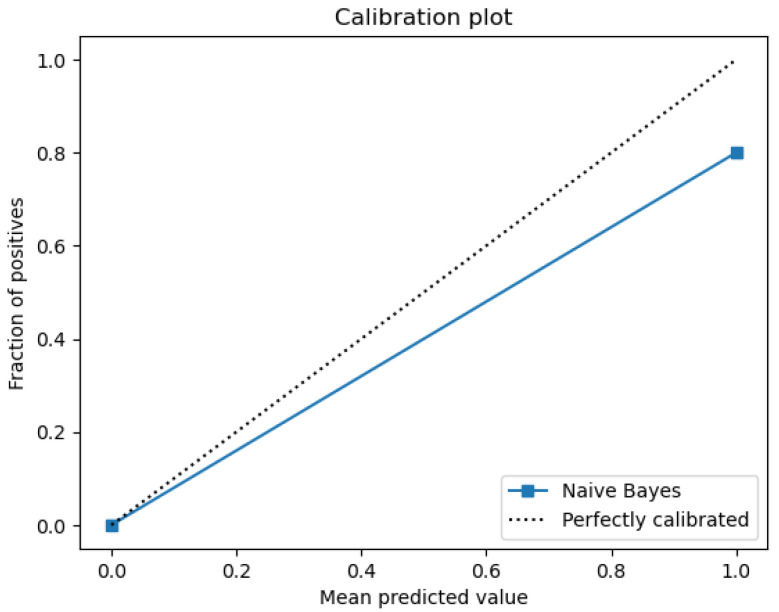
Naïve Bayes: calibration plot.

The plots that are presented in the figures below showcase the naïve Bayes classifier results for water quality data. Figure 11 represents the ROC curve, reflecting how accurately the model can distinguish drinkable from non-drinkable samples of water. Figure 12 represents a precision–recall plot, and Figure 13 is a calibration plot reflecting how reliable the estimated probabilities are.

The naïve Bayes classifier performs with almost the same level of accuracy as logistic regression, with about 83% of water samples being classified correctly. Its AUC at 0.99 indicates an excellent discriminant power, with its precision-recall curve exhibiting practically no fluctuations at all. Its precision-recall curve’s naïve Bayes calibration plot leans towards a diagonal, rather than toward logistic regression, indicating that its probabilities for predictions are closer to true probabilities, although not fully calibrated.

### 4.4. Decision Tree

**Figure 14:**
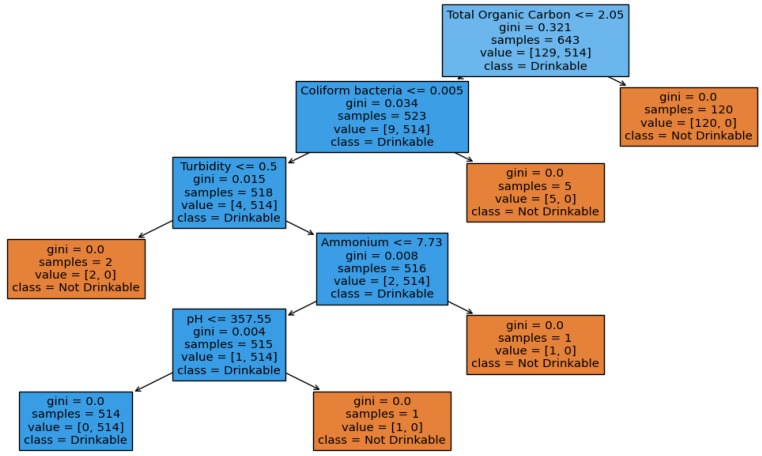
Decision Tree plot.

**Figure 15:**
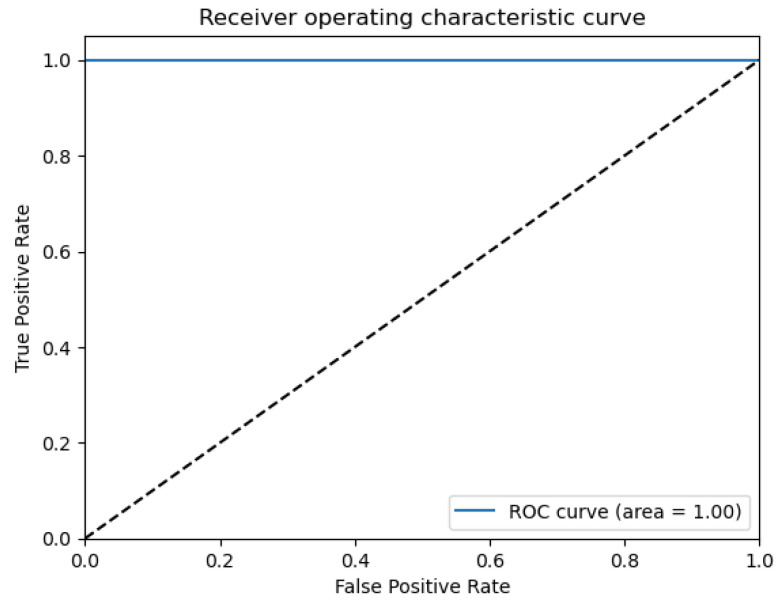
Decision Tree: ROC curve, where the dashed line represents Random guess.

**Figure 16:**
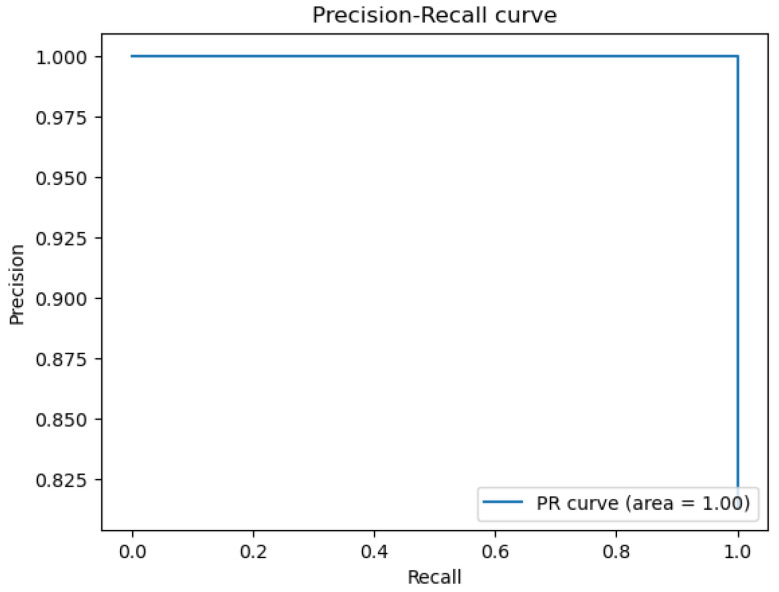
Decision Tree: precision–recall curve.

**Figure 17:**
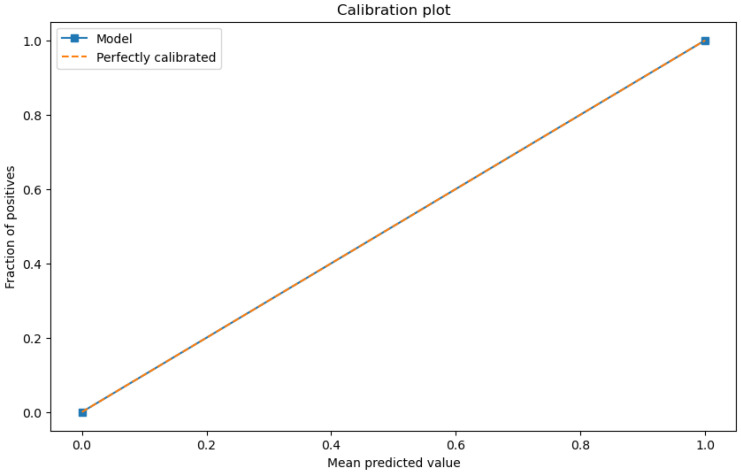
Decision Tree: calibration plot.

The figures below provide a clear description of the way in which the decision tree model performed on the water quality dataset. Figure 14 shows the tree structure for classification. Figure 15 shows the ROC curve, Figure 16 indicates the precision-recall, and Figure 17 shows the probability calibration of the model.

Figure 14 presents the decision tree learned from the existing water quality data. The model arrives at a final decision in six levels of depth. In performance, the decision tree performs perfectly, achieving an accuracy of 1.00, with an ideal ROC, as well as precision-recall curves, as presented in Figure 15 and Figure 16. The implemented decision tree classifier achieved perfect accuracy related to the test set. This result reflects the strong separability characteristic of the acquired dataset, in which potable and non-potable labels are also defined through the regulatory threshold exceedance of physicochemical parameters. Under these specific conditions, the decision tree recovers deterministic decision rules embedded in the data. Consequently, the derived perfect accuracy is an indicator of internal consistency, rather than evidence of a broad generalization capability, but it also enables an automated representation of regulatory logic to be further applied to large-scale monitoring scenarios. The calibration plot (Figure 17) verifies that its predicted probabilities perfectly agree with outcomes that are observed. The total organic carbon (TOC) offers a contribution of about 0.9, which is several times larger than the contribution of other features. This aligns with prior domain knowledge, as high TOC content might indicate the presence of toxic organic pollutants like pesticides, industrial contaminants, or natural organic content, which notably influence water potability.

### 4.5. Random Forest

**Figure 18:**
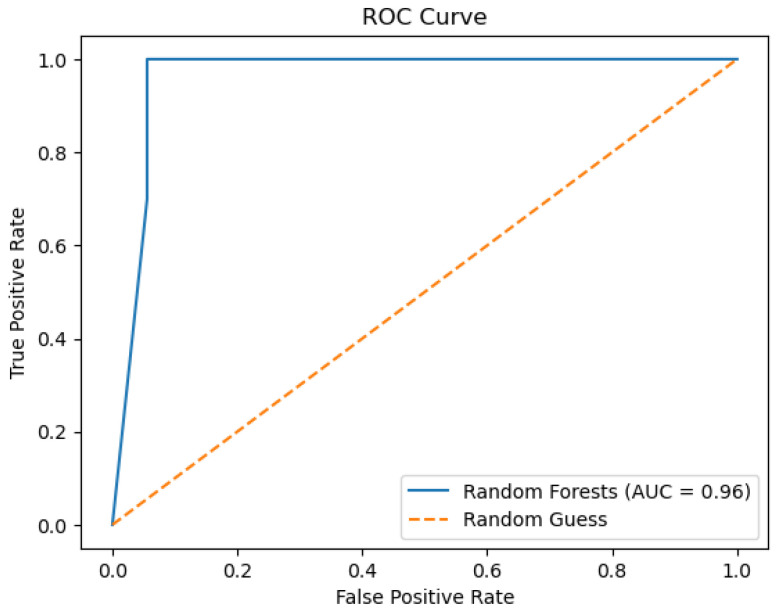
Random Forest: ROC curve.

**Figure 19:**
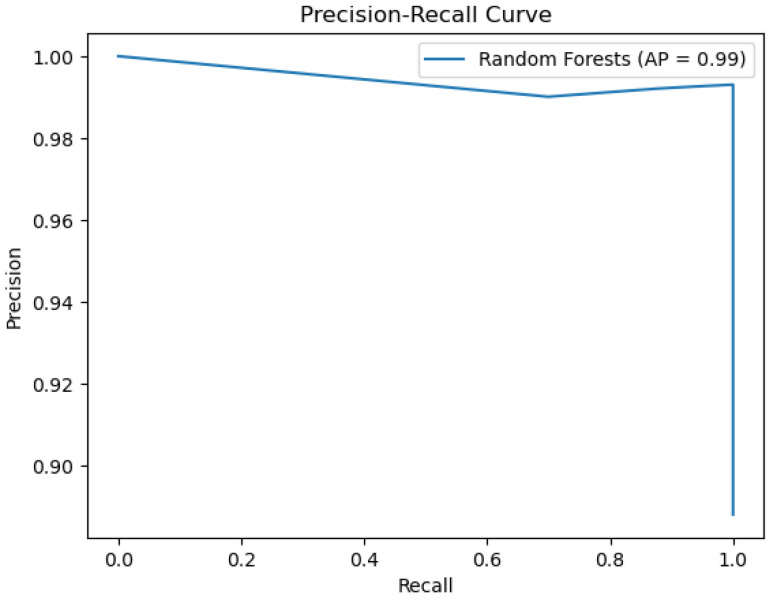
Random Forest: precision–recall curve.

**Figure 20:**
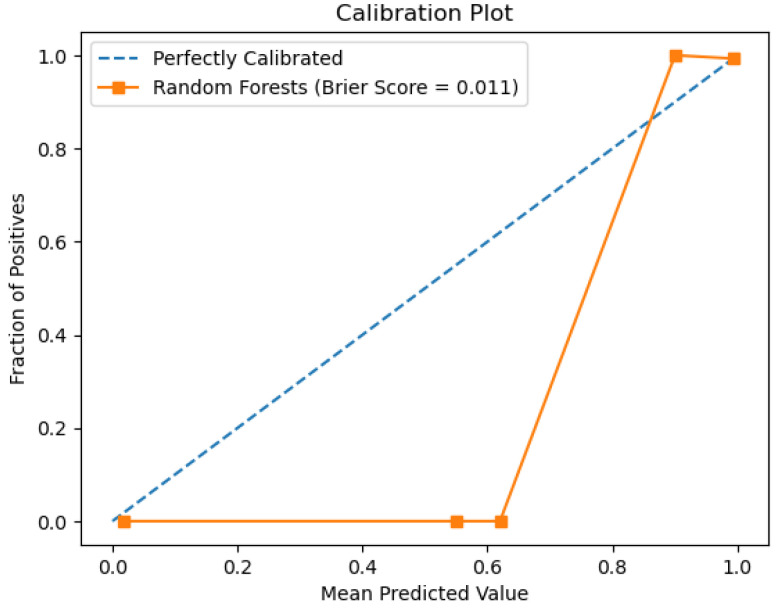
Random Forest: calibration plot.

The random forest model’s performance is depicted below in the figures. Figure 18 shows the ROC curve, whereas Figure 19 depicts the precision–recall plot. Figure 20 shows the model’s behavior.

The plots above represent the results of the random forest classifier on the existing data regarding water quality. The classifier’s performance, as represented by an accuracy of 0.98 and an AUC score of 0.96, indicates good overall classifier results. There are non-ideal deviations from the diagonal for this model’s Calibration Plot, meaning that probability estimates were not well calibrated, unlike in more reliable calibration with the decision tree algorithm. When it comes to feature importance, as for earlier models, total organic carbon remains the most dominant predictor, although, for other variables, there is a minor contribution towards decision-making.

### 4.6. Support Vector Machine

**Figure 21:**
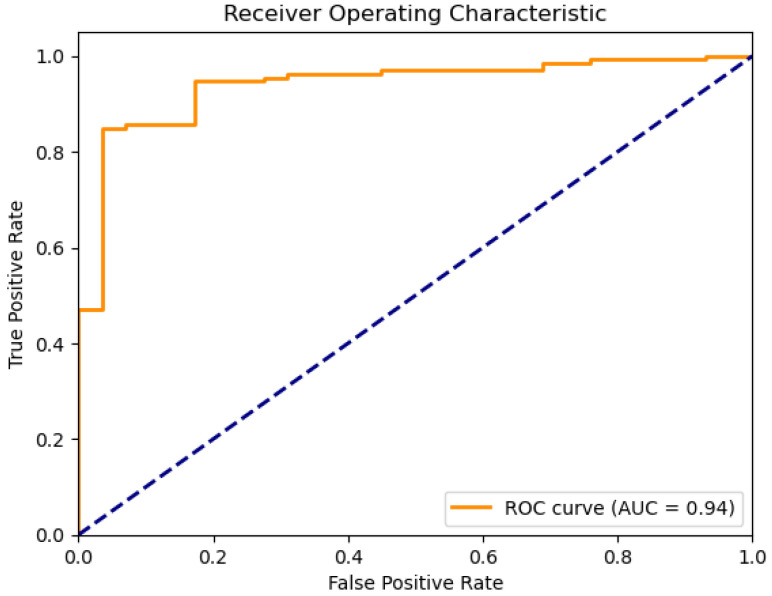
SVM: ROC curve, where the dashed line represents Random guess.

**Figure 22:**
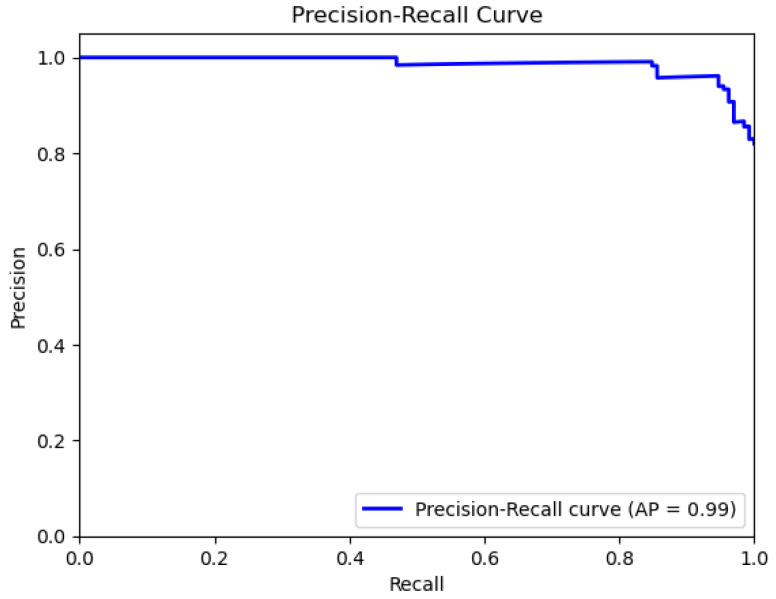
SVM: Precision–recall curve.

**Figure 23:**
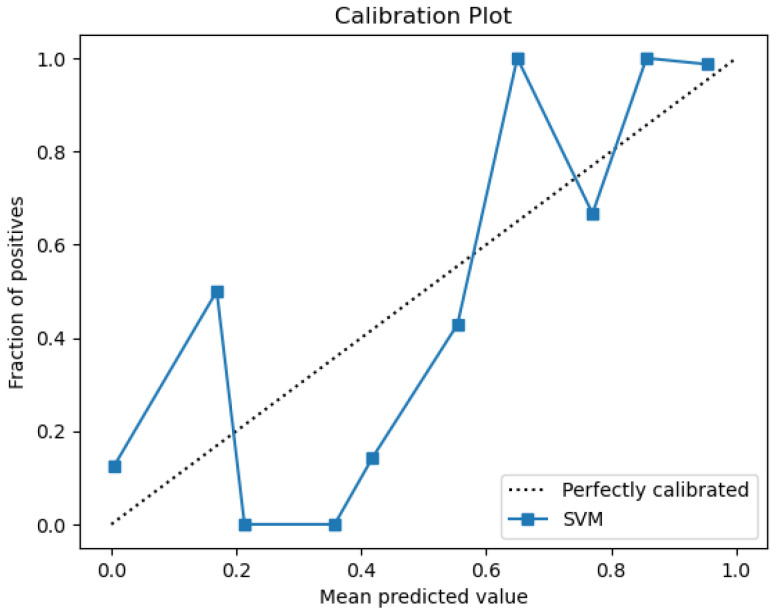
SVM: Calibration Plot.

The following plots depict the outcome of implementing the Support Vector Machine (SVM) model in classifying water quality. Figure 21 presents the ROC curve, which reveals the discriminative ability of the model, Figure 22 presents the precision–recall relation, and Figure 23 presents the calibration curve, which reveals information about the concordance between predicted probabilities and true outcomes.

Support Vector Machines (SVM) find optimal hyperplanes in high-dimensional spaces for the best classification. For this water dataset, 81% accuracy with a good AUC value of 0.94 was achieved, showing good discriminative power. However, as the calibration plot shows (Figure 23), there are erratic spikes, which suggest instability in probability estimation.

The SVM model is less precise compared to the algorithms presented earlier.

### 4.7. K-Nearest Neighbors

**Figure 24:**
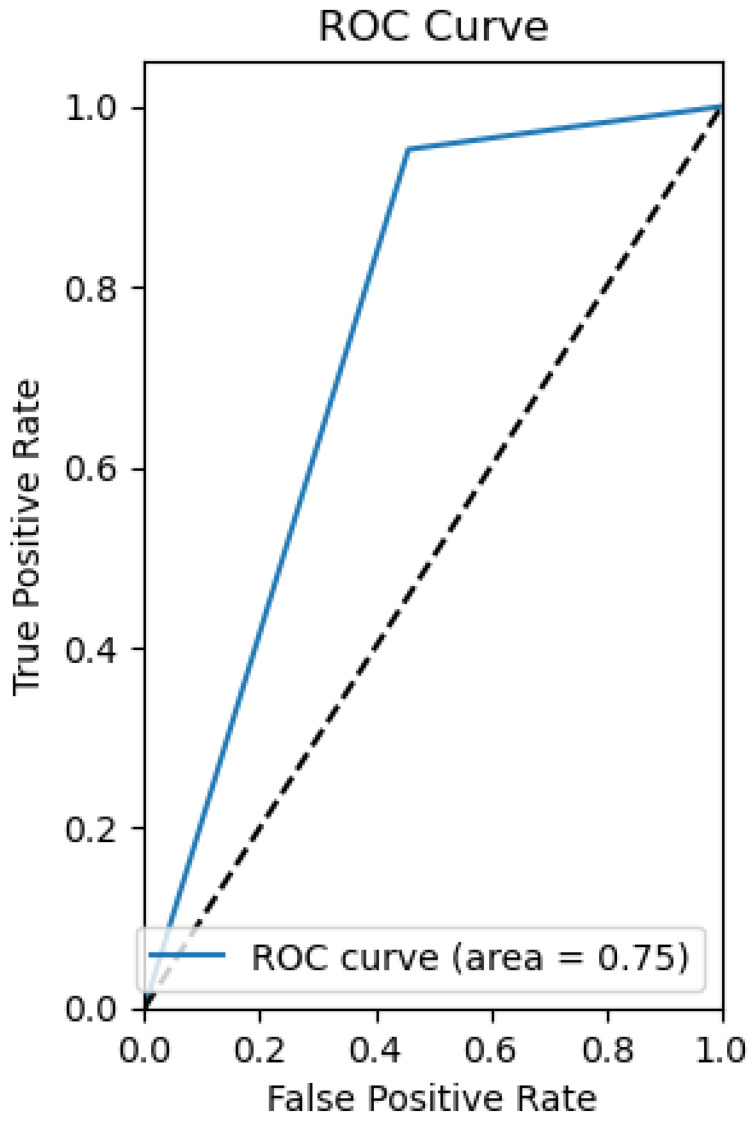
KNN: ROC curve, where the dashed line represents Random guess.

**Figure 25:**
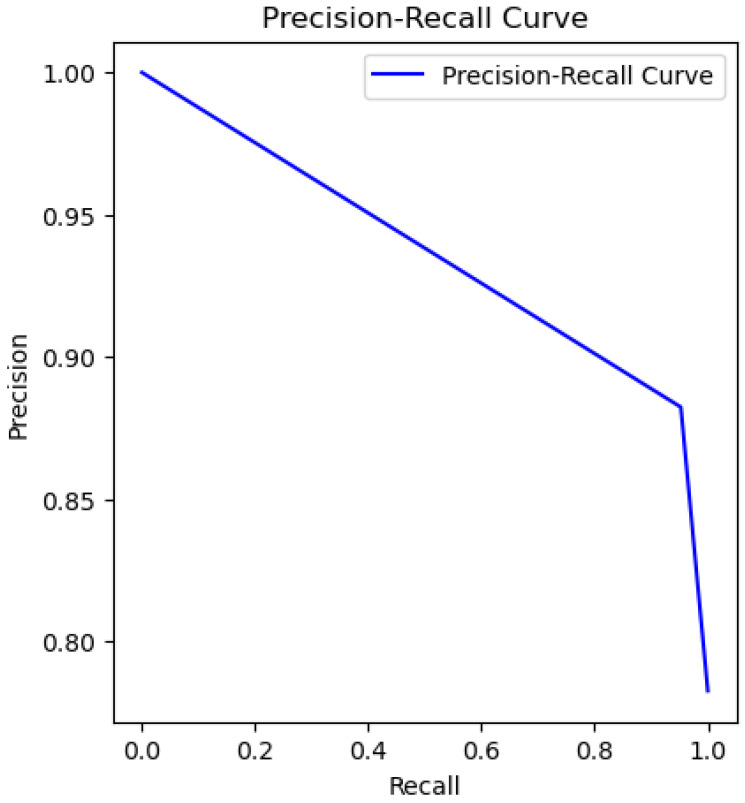
KNN: precision–recall curve.

**Figure 26:**
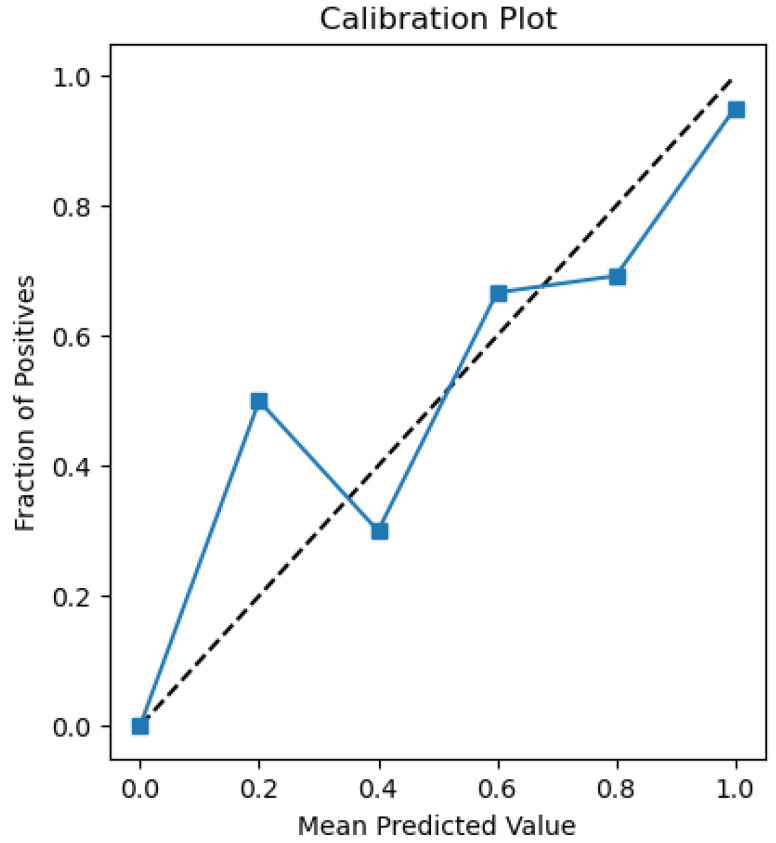
KNN: calibration plot in blue where the dashed line represents perfect calibration.

**Figure 27:**
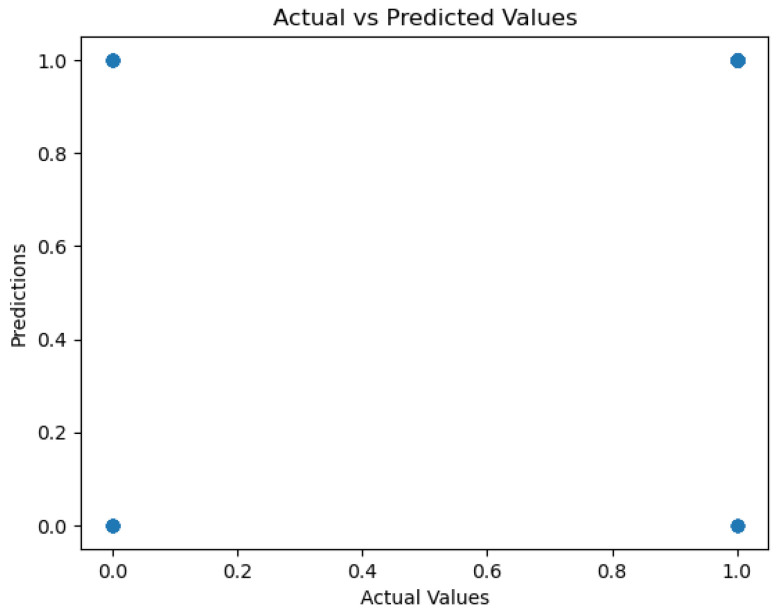
KNN: actual vs. predicted values.

The plots presented below show the K-Nearest Neighbors (KNN) model’s performance across multiple evaluation metrics. Figure 24 demonstrates the ROC curve, and Figure 25 exhibits the precision–recall relation. In Figure 26, the model calibration is presented, while in Figure 27, a comparison between predicted and actual values is shown. Collectively, the plots present a complete picture of a model’s predictive behavior.

The water quality classification KNN attained 86% accuracy, meaning that most samples were properly classified as either drinkable or non-drinkable. Nevertheless, its general discriminability is subdued, with the ROC area being 0.75. The precision–recall curve starts at ideal precision but drops to 0.90, indicating that, although initially drinkable water samples are properly classified, the probability of missing positive instances increases as the recall threshold increases. The calibration plot results in multiple irregularities; the probabilities predicted are unreliable, compromising a probabilistic interpretation for the model. Lastly, the Actual vs. Predicted Values graph (Figure 27) exhibits both outliers and misclassification, further validating the finding that, for this application, the KNN model, though relatively successful, shows various performance weaknesses.

### 4.8. Ada Boost

**Figure 28:**
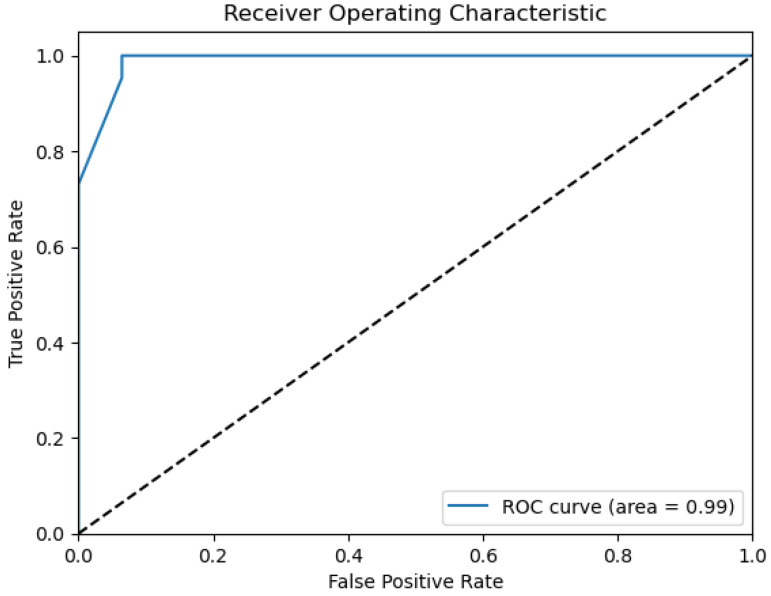
AdaBoost: ROC curve where the dashed line represent Random guess.

**Figure 29:**
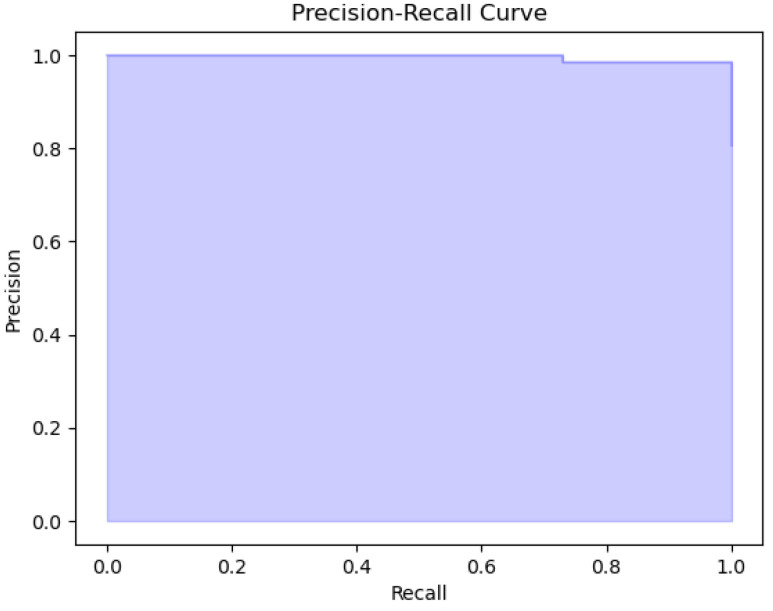
AdaBoost: precision–recall curve.

**Figure 30:**
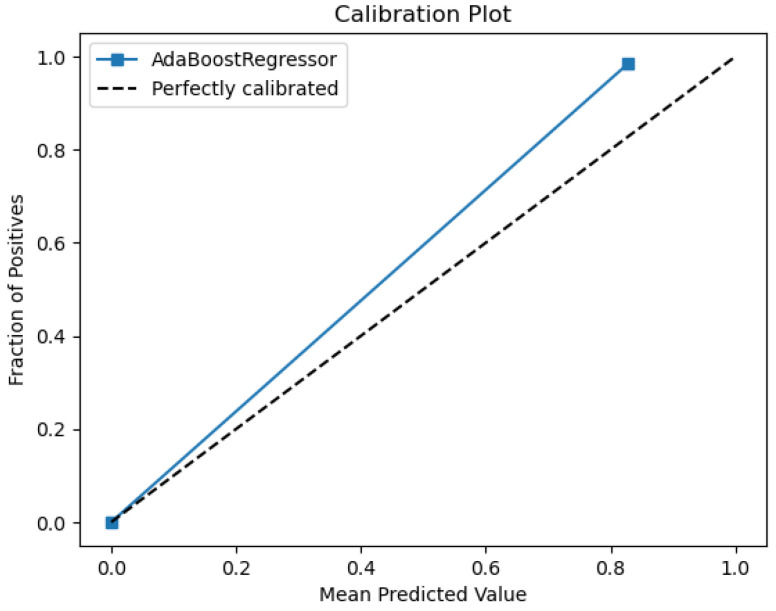
AdaBoost: Calibration Plot.

**Figure 31:**
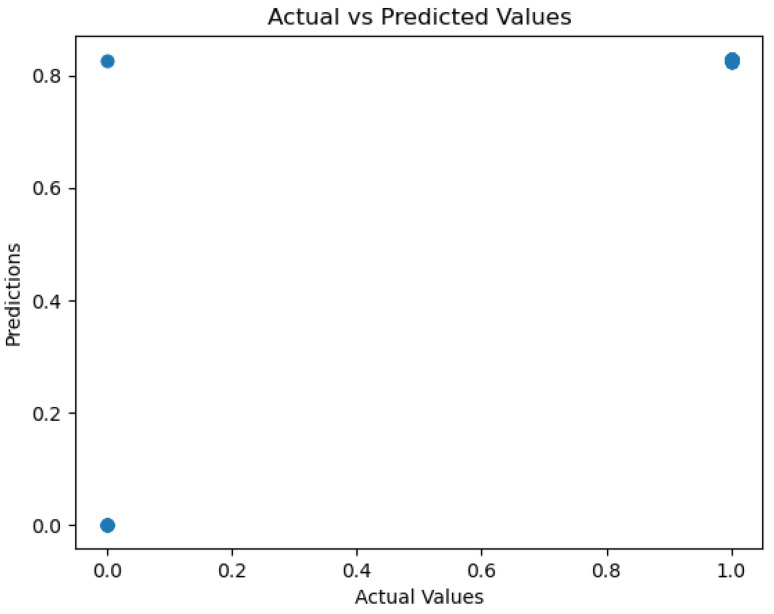
AdaBoost: actual vs. predicted values.

**Figure 32:**
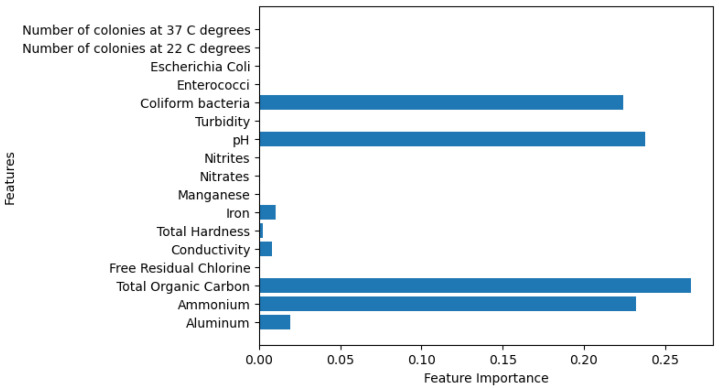
AdaBoost: feature importance.

Figure 28, Figure 29, Figure 30, Figure 31 and Figure 32 contain the most relevant metrics and parameters used by the model.

AdaBoost also provides an ROC area of 0.99 for water quality and demonstrates an excellent categorization capability between drinkable and non-drinkable water samples. The precision–recall curve that corresponds to it exhibits a perfectly precise first phase, dropping slightly afterwards with rising recall. This implies that, although the model performs extremely well in picking out positive (drinkable) cases, it will, at times, wrongly classify samples at more liberal recall thresholds. The calibration plot (Figure 30) first plots close to an ideal diagonal, but a minimal deviation manifests at high probabilities, which implies minimal overconfidence in its predictions. This implies that, although generally properly calibrated, estimated probabilities of the model rarely exactly equal observed outcomes.

The relationship between actual and predicted values is presented in a plot indicating how the AdaBoost model showcases a highly confident level of predictions. The plot indicates possible instances of faulty classification, sometimes generating overconfident predictions. By utilizing a calculated Mean Squared error of 0.032, the model showcases powerful predictive abilities. Its R^2^ score of 0.7918 shows good performance. The proposed model’s predictions are influenced by four variables, namely total organic carbon (TOC), ammonium, pH, and *Coliform bacteria*. While, again, TOC stands out as the most dominant, the other three features exhibit an almost equal influence over the model’s decision-making, as shown in Figure 32.

### 4.9. Gradient Boosting

When trained with the water quality dataset, gradient boosting performs better than AdaBoost, with an ROC area of 0.99 and a practically perfect precision–recall curve. The calibration plot lies almost perfectly on the ideal diagonal, which shows that predicted probabilities are properly calibrated as a good indication of true probability for each class. The accuracy of 99.6% presented in the actual vs. predicted plot highlights the model’s outstanding ability to predict water’s potability.

**Figure 33:**
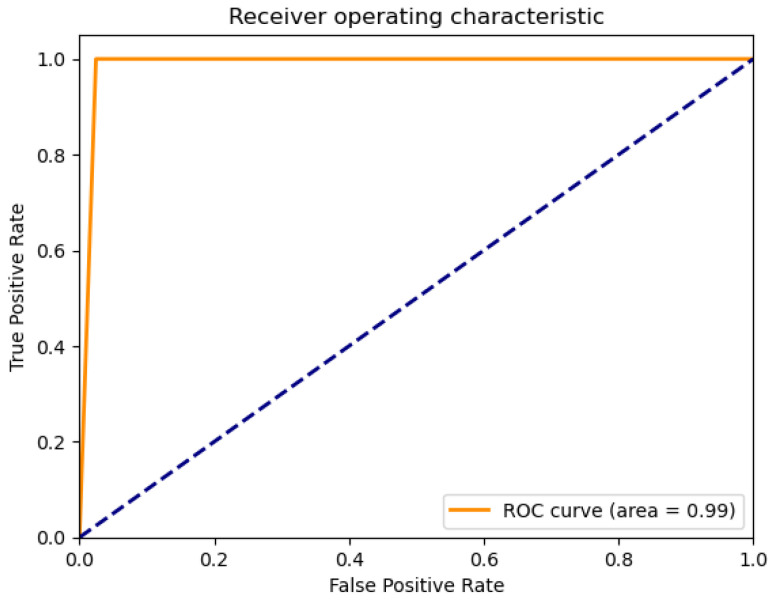
Gradient Boosting: ROC curve where the dashed line represents Random guess.

**Figure 34:**
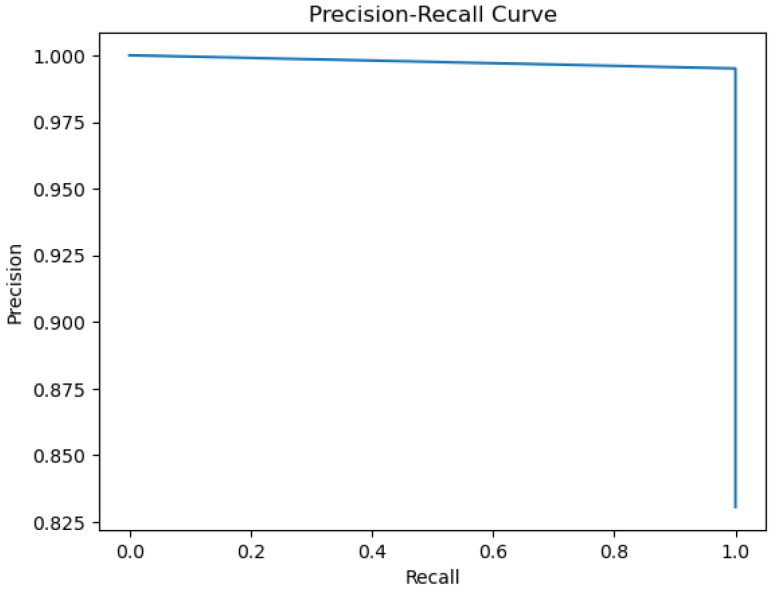
Gradient Boosting: precision–recall curve.

**Figure 35:**
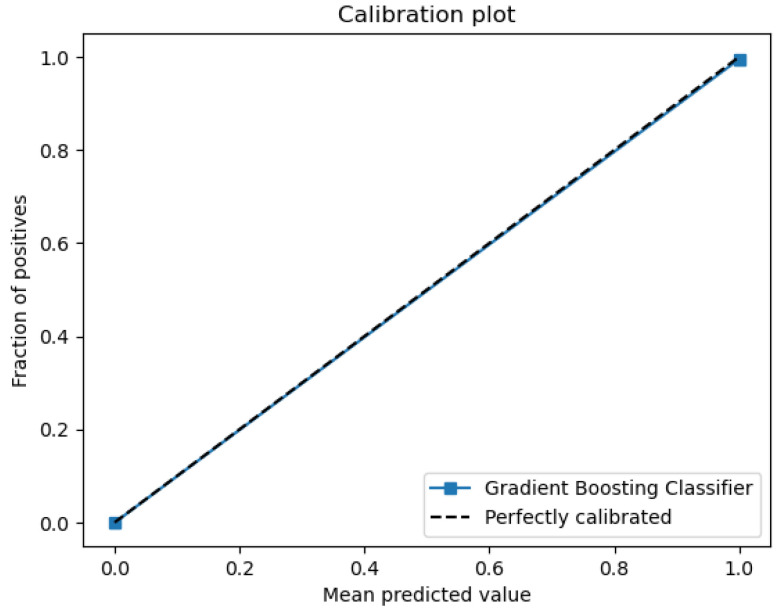
Gradient Boosting: calibration plot.

**Figure 36:**
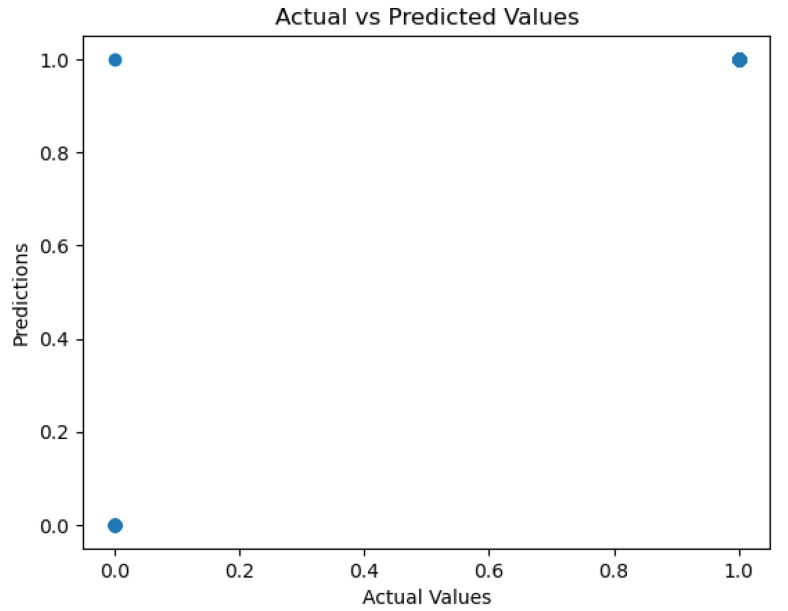
Gradient Boosting: actual vs. predicted values. Each dot represents a test sample, where the x-axis corresponds to the true class and the y-axis to the model’s predicted class.

**Figure 37:**
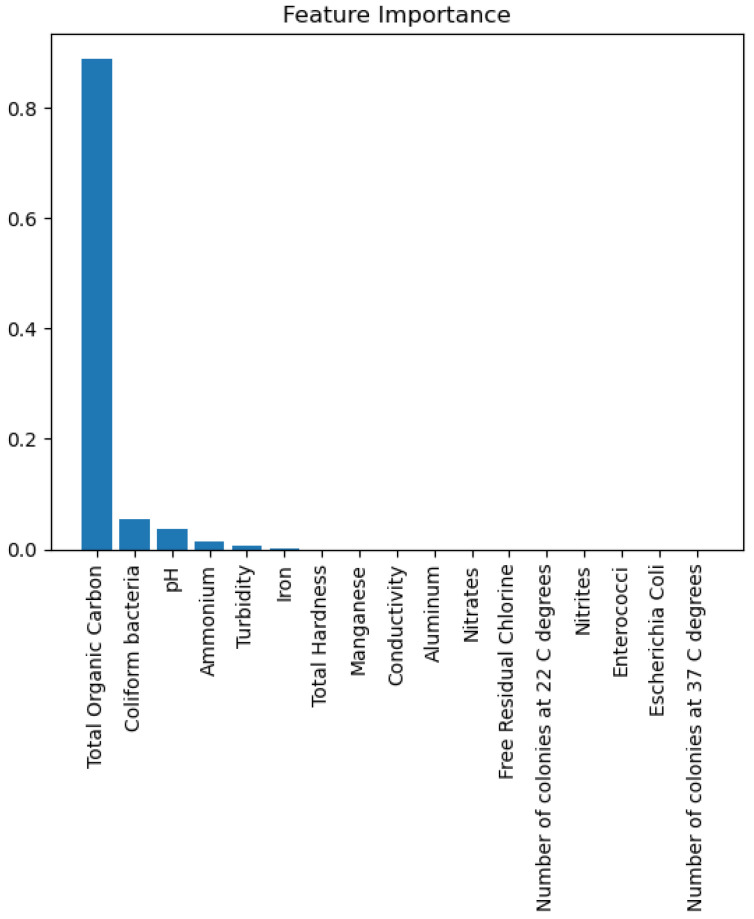
Gradient Boosting: feature importance.

The metrics could be viewed in Figure 33, Figure 34, Figure 35, Figure 36 and Figure 37.

The gradient boosting model utilizes the same four major variables as before: total organic carbon (TOC), pH, ammonium, and *Coliform bacteri*. TOC is still the dominant variable, as shown in Figure 37, where we can observe an importance score almost equal to 0.9. The gradient boosting model achieves an overall very good performance for all metrics of evaluation.

### 4.10. Bagging Classifier

**Figure 38:**
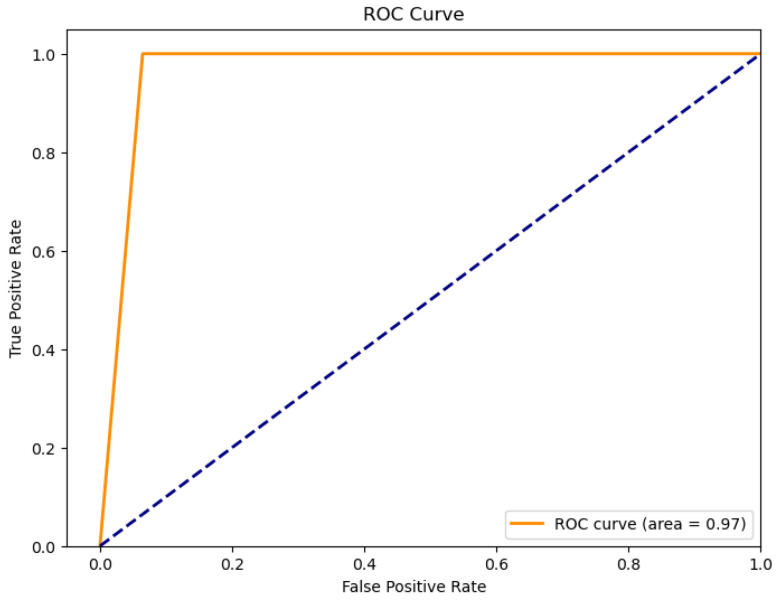
Begging Classifier: ROC curve where the dashed line represents Random guess.

**Figure 39:**
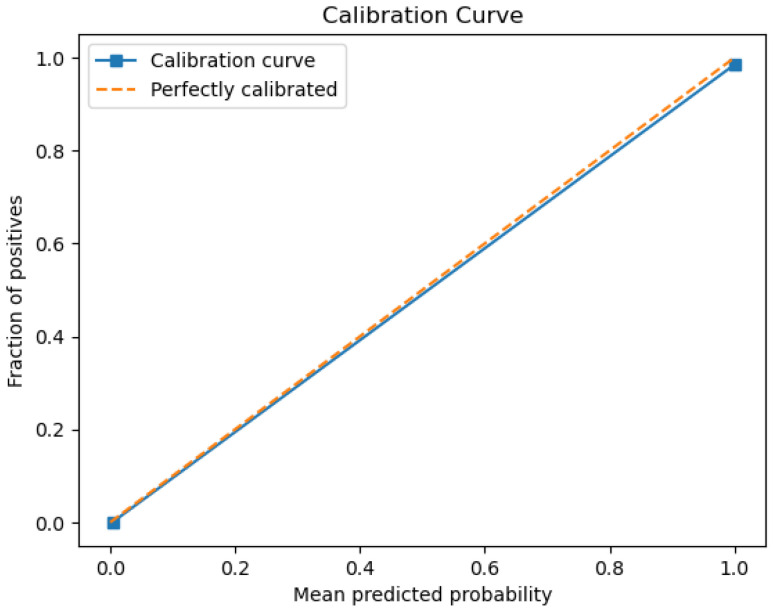
Begging Classifier: scatter plot for training data.

**Figure 40:**
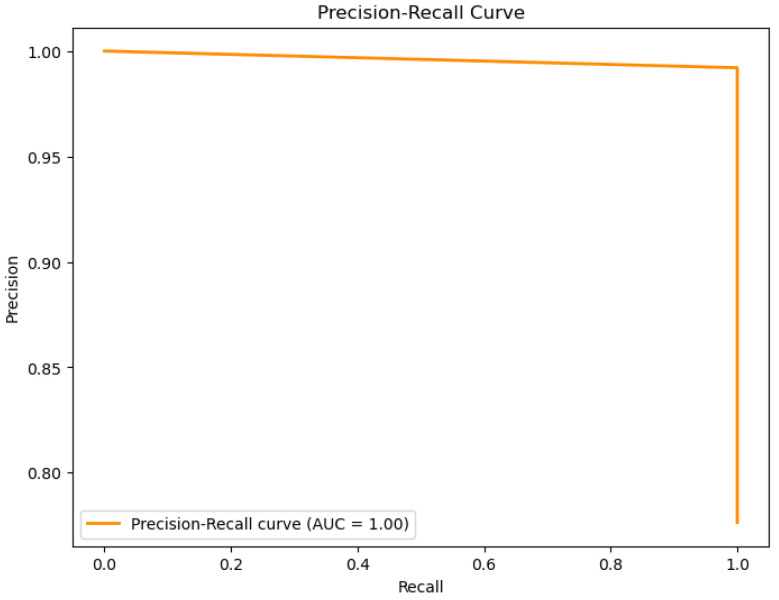
Begging Classifier: calibration plot.

**Figure 41:**
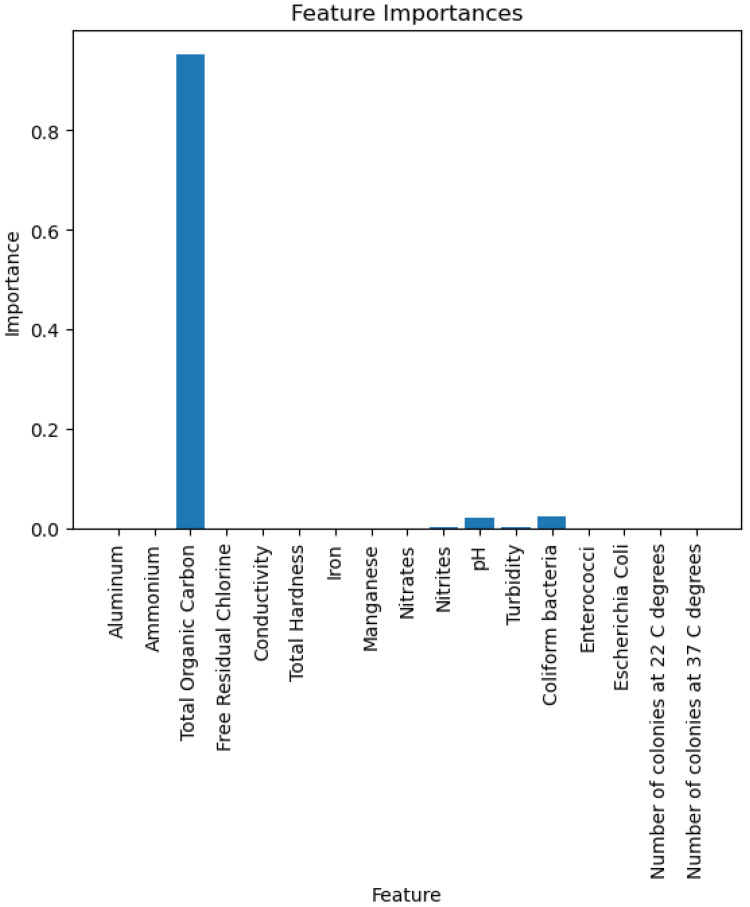
Begging Classifier: scatter plot for testing data.

In application to the water quality data, the ROC area of 0.97 indicates that the Bagging Classifier performs approximately as well as the gradient boosting model. The value of the ROC area is 0.97, indicating that the Bagging Classifier is performing at almost the same precision levels as the gradient boosting model. The precision–recall curve has also attained perfection, having very high discriminative power and good levels of trustworthiness. Only a small deviation below the ideal diagonal appears on the calibration plot, validating the proper calibration of the model’s estimates. The classifier performs with an accuracy of 98.8%, which falls about 1% below that of gradient boosting. Figure 41 indicates that, again, total organic carbon (TOC) continues as the highest influential parameter in watermark potability prediction. All significant parameters that contribute to the output are given in a graph in Figure 38, Figure 39, Figure 40 and Figure 41.

### 4.11. Linear Discriminant Analysis

**Figure 42:**
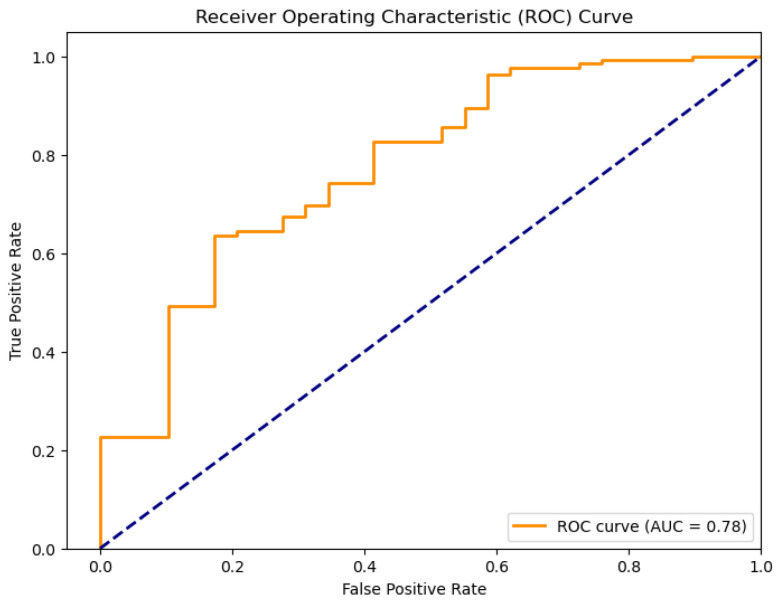
LDA: ROC curve where the dashed line represents Random guess.

**Figure 43:**
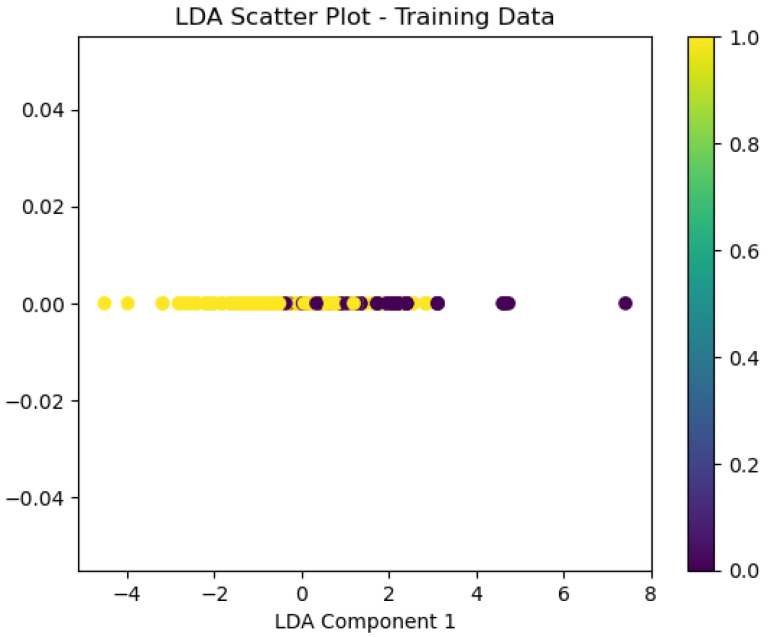
LDA: scatter plot for training data.

**Figure 44:**
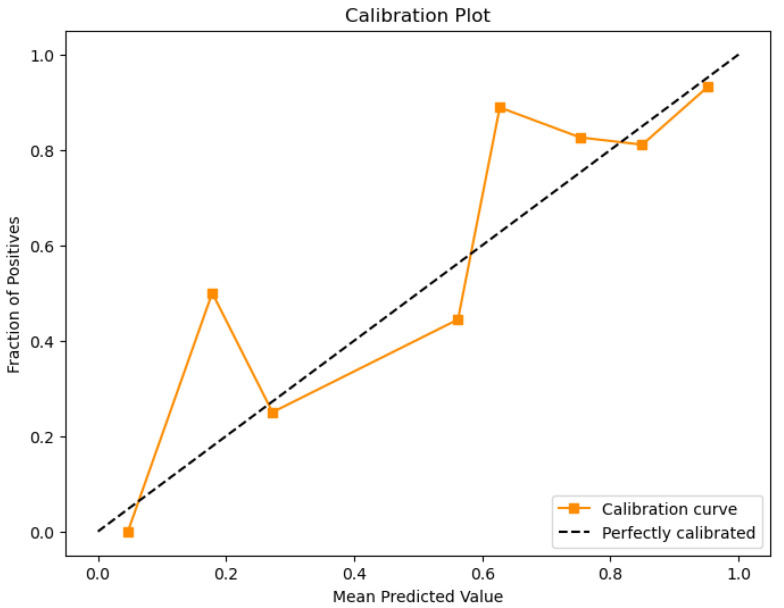
LDA: calibration plot.

**Figure 45:**
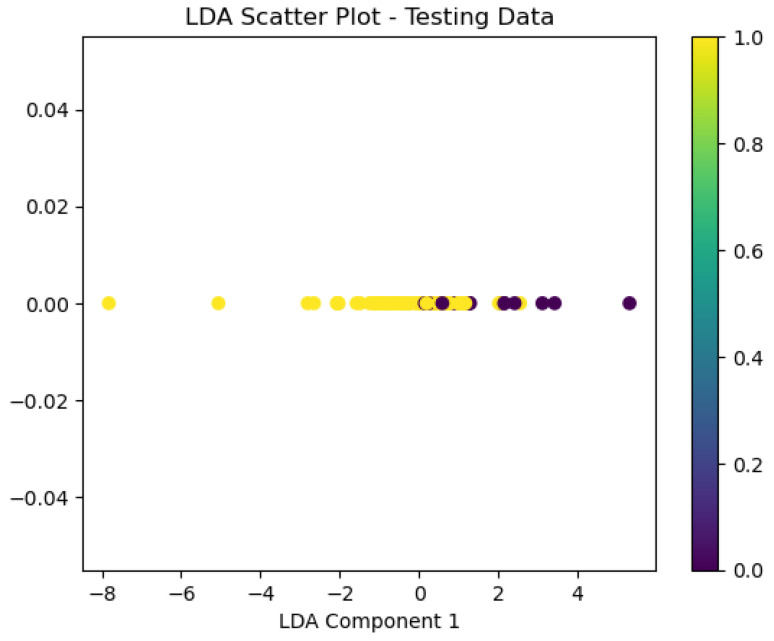
LDA: scatter plot for testing data.

For the Timișoara water quality dataset, LDA exhibited moderate predictive accuracy for how potable the water was, as is presented below in Figure 42, Figure 43, Figure 44 and Figure 45.

The obtained value of a 0.78 ROC area suggests moderate classification performance, while the scatter plot means the model exhibits minimal class separation. The calibration plot (Figure 44) indicates significant fluctuations, which means the model lacks uniformly calibrated probability estimates.

### 4.12. Multilayer Perceptron

For this experiment, an MLP was coded via Keras in order to compare its efficiency for predicting water potability with a series of physicochemical parameters.

**Figure 46:**
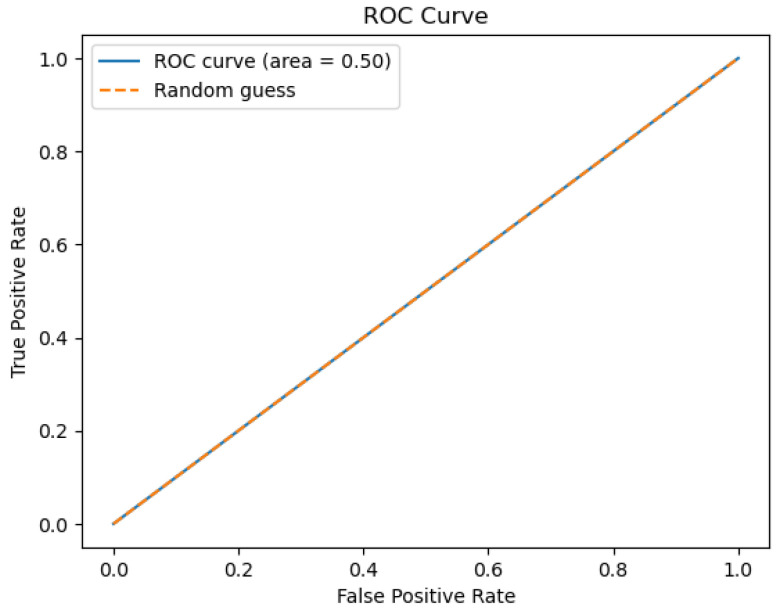
Neural Networks: ROC curve where the dashed line represents Random guess.

**Figure 47:**
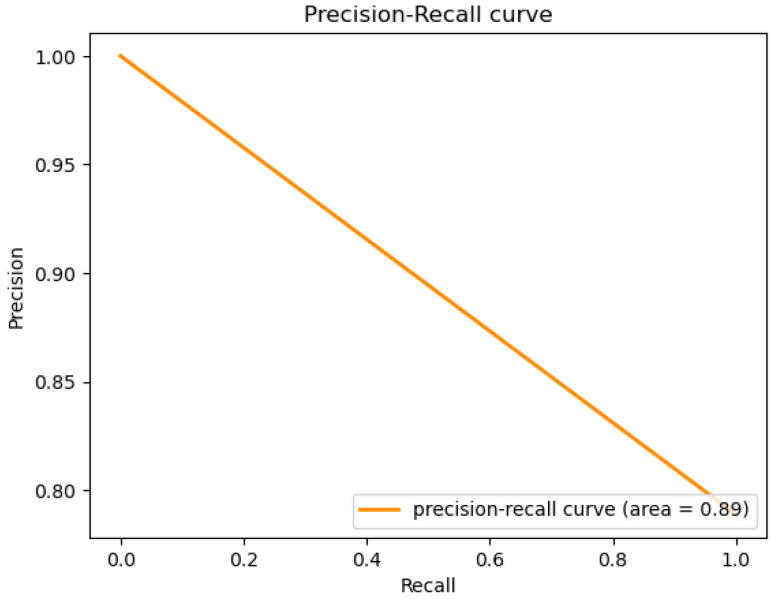
Neural Networks: precision–recall curve.

**Figure 48:**
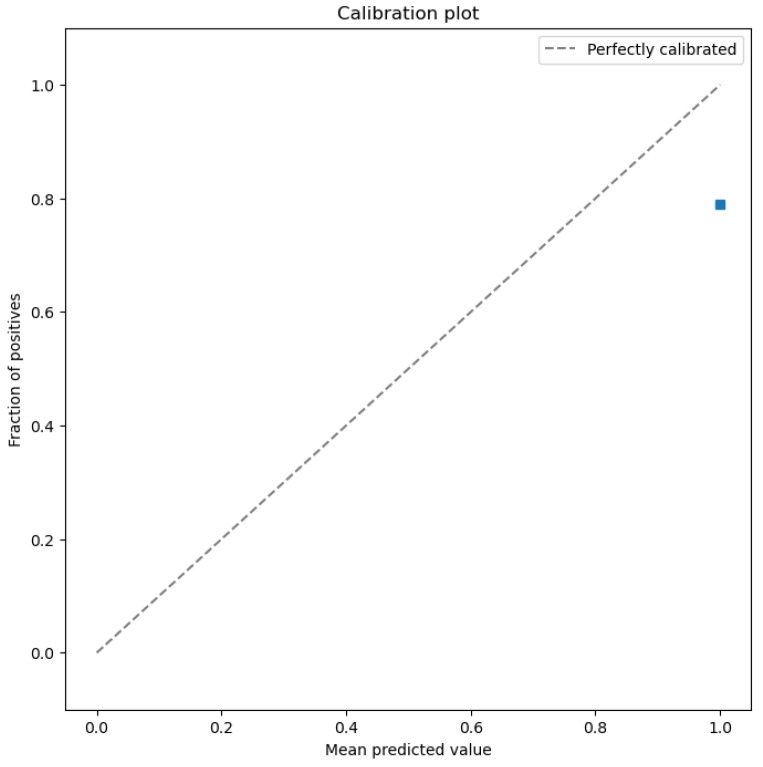
Neural Networks: calibration plot where the dashed line represents perfect calibration.

**Figure 49:**
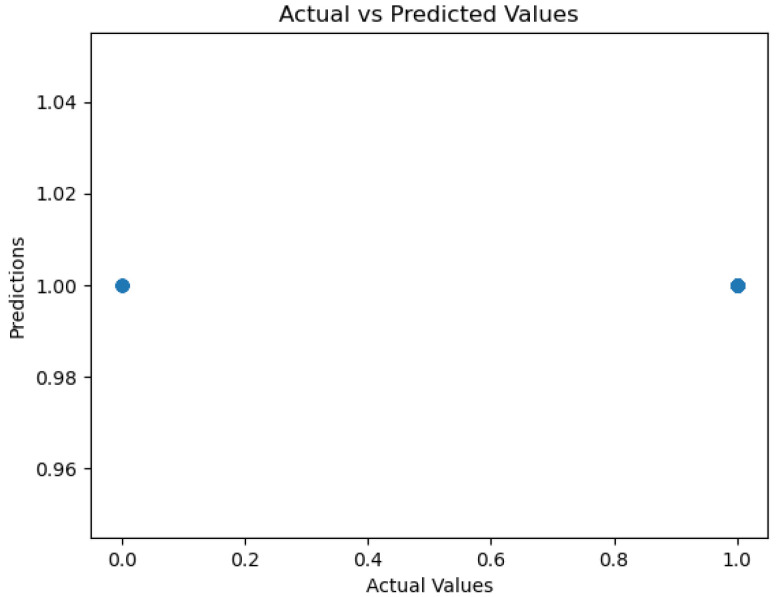
Neural Networks: actual vs. predicted values. Each dot represents a test sample, where the x-axis corresponds to the true class and the y-axis to the class predicted by the neural network model.

For the neural networks of this proposed system, the Keras Python library was employed; it is a high-level deep learning framework offering a convenient interface for building, as well as training neural networks. For existing data, for example, in the case of predicting water drinkability, the results are given in Figure 46, Figure 47, Figure 48 and Figure 49.

The ROC area depicted in Figure 46, with a value of 0.5 implies that the performance of the model in separating drinkable and non-drinkable water samples equals that of random guessing. This indicates how the model is missing significant patterns in the data, which are needed for a trustworthy classification. The precision–recall curve area measures 0.89 instead, showing moderate performance in trading off precision for better recall. The related calibration plot confirms a poor calibration level of the predicted probabilities of the model, potentially resulting in overconfident or underconfident predictions.

**Figure 50:**
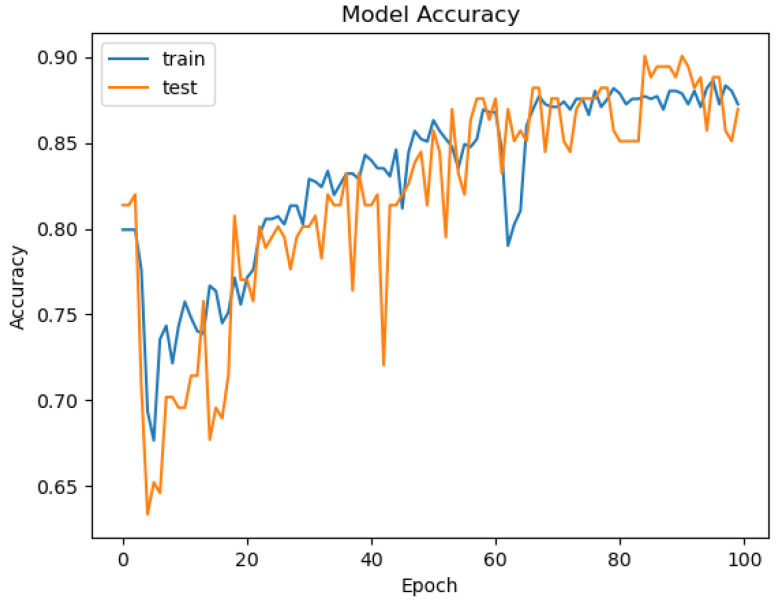
Neural Networks: model accuracy.

The accuracy plot that we present in Figure 50, varying between 0.65 and 0.9 over the epochs, highlights an inconsistency in the performance of the model and potentially indicates a sign of instability and overfitting. A steady decline over time for model loss indicates instead a decrease over time from about 17.5 to near 0 by epoch 20, suggesting learning and optimizing over time. In spite of such training loss improvements, other assessment metrics are a concern. A low value of ROC AUC, a decreasing precision–recall curve, and the inconsistencies in the calibration indicate serious weaknesses in the predictions of the model. Furthermore, the error histogram shows a disproportionate misclassification for non-drinkable samples (class 0) with a peak over 120, compared to a lower error count for drinkable samples (class 1), with a peak at about 40. The presented outcomes indicate that the Keras neural network model might underperform when predicting water potability with the dataset and feature range presented.

**Figure 51:**
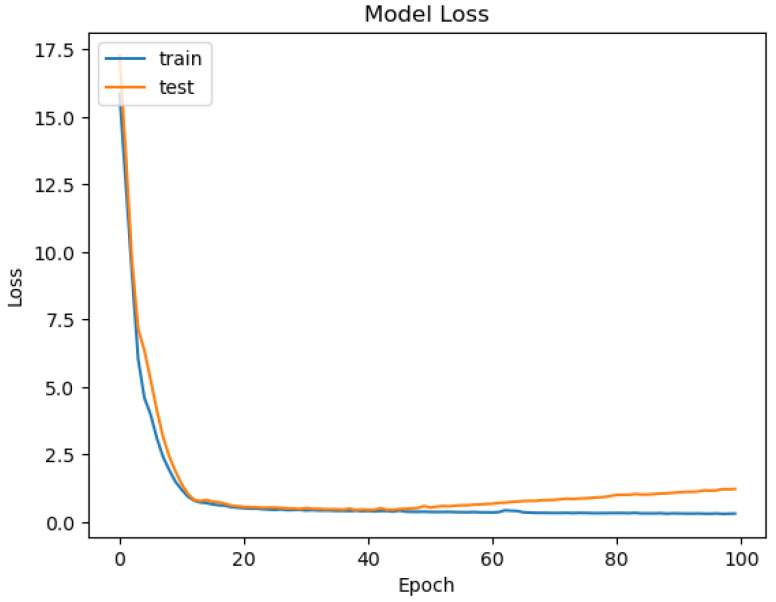
Neural Networks: model loss.

**Figure 52:**
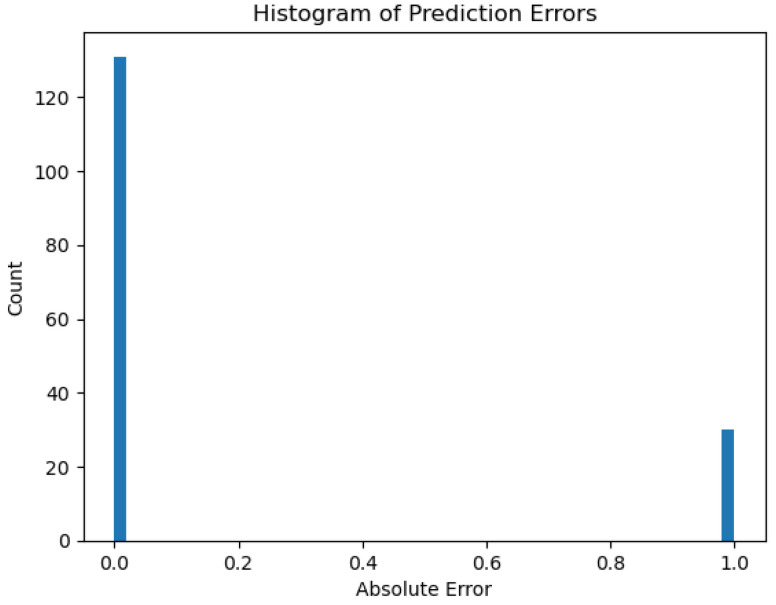
Neural Networks: histogram of prediction errors.

In addition, we depict in Figure 51 the loss curve of the model that indicates neural network learning during training, and in Figure 52, we reveal the error histogram in predictions that shows classification accuracy mismatches between both output classes.

## 5. Discussion

A summary of the presented results of the ML algorithms for water drinkability prediction is presented in Table 1 where "–" indicates the fact the accuracy metric and the PRC Area were not available for the MLP (Keras) and the Linear regression models, respectively. The MLP (Keras) model output probabilities were not converted into discrete class labels. Linear Regression also produces continuous regression outputs, not probabilistic classification predictions that would be required for Precision–Recall analysis.

Achieving 100% accuracy and perfect calibration was possible with the decision tree model in the Timișoara Public Water Network dataset. Other ensemble models, such as random forest, gradient boosting, and Bagging Classifier, achieved high accuracy and perfect calibration. However, it was found that the decision tree model was ranked as the best-performing and most suitable model in achieving potability classification according to WHO standards.

From the water quality data provided in the Timișoara Public Water Network dataset, some conclusions can be drawn. The major highlights of what can be concluded from the data are presented in Table 2. Of special interest is that the best model in drinkability prediction is determined, and the recommendations that can be made based on the data are presented. Although these results are dynamic and may change based on new samples and changes in the public water network, it can be seen that these results are robust and reliable.

In the Timișoara Public Water Network dataset, it can be seen that there is a dominant class in drinkability. The decision tree model was found to be reliable in achieving potability prediction. The results of this study are supported by the recommendations based on the results of the study. To further validate this study, it can be seen that this study was compared with other machine learning models based on other studies in the literature. Seven studies from the reference list are compared with this study in achieving drinkability prediction. The results are shown in Table 3.

The results of this study are supported by other decision tree models in river and drinking water quality prediction [21,24,25] and other models, such as LightGBM and KNN, in other studies [23,26]. This study achieved perfect classification accuracy in contrast with other machine learning models in achieving drinkability prediction. This study is different from other studies in that it used actual data from a public water network in Timișoara.

Finally, the results indicate the strong performance of decision tree models for use cases related to water potability, especially for tasks that involve meeting critical threshold values. Last but not least, decision tree models support interpretability by enabling a clearer understanding of filtration and decision processes, in contrast to neural networks, which generally lack transparency and are less interpretable.

### 5.1. Feature Crafting and Data Preparation

In this regard, feature selection for the machine learning models was carried out using a domain-guided approach, for which the primary objective was to evaluate the potability of water in alignment with World Health Organization (WHO) standards. The parameters selected for this purpose include various physicochemical and microbiological properties that are typically monitored in public water distribution systems and have been identified in the literature as essential factors influencing drinking water quality.

To this end, parameters such as pH, turbidity, nitrate content, total organic carbon (TOC), etc., have been selected because they are specifically addressed through essential threshold values set by the WHO. Exceeding these threshold values directly implies a threat to public health. Therefore, these features have a unique physical interpretation that directly correlates with their operational implications.

In this regard, feature transformation for this machine learning-based approach has not been carried out using sophisticated statistical models such as dimensionality reduction or feature learning. Instead, the features have been directly used from their measured values, in which the classification labels have been directly mapped based on compliance with WHO threshold values. This was undertaken deliberately to ensure experimental reproducibility, the transparency of the decision-making processes, and alignment with decision tree models that are inherently suitable for threshold-based classification problems.

### 5.2. Evaluation of Model Performance Using Safety-Oriented Metrics

In order to assess the reliability of the classification models from the perspective of the safety of drinking water, the performance analysis of the models has been further extended to include precision, recall, F1 score, and confusion matrices. This is important in the context of public health, where the false negative errors that may result in the misclassification of non-drinkable water samples as drinkable may pose a direct risk.

#### 5.2.1. Performance of the Decision Tree Model

The decision tree model was tested on a test set consisting of 161 water samples, out of which 129 samples were drinkable, and 32 samples were non-drinkable, as shown by the performance of the model below:

- Precision = 1.00.
- Recall = 1.00.
- F1-score = 1.00.
- Overall accuracy = 1.00.

The results for both macro-average and weighted-average values show that their results were all 1.00. The results for the confusion matrix also show that there were no classification errors, as no false negatives or false positives were recorded. This demonstrates that the decision tree model can correctly classify all non-drinkable water samples without any of them being classified as drinkable, making it an ideal model for safety-critical applications. This demonstrates that it can correctly recover deterministic relationships as defined by threshold exceedance.

#### 5.2.2. Performance of the Bagging Classifier

The Bagging Classifier also achieved very high performance, with an accuracy of 0.9938. The evaluation was conducted on the same test set of 161 samples, and the resulting confusion matrix was:

$$\left[\begin{array}{cc} 35 & 1\\ 0 & 125 \end{array}\right]$$

The corresponding classification report indicates the following values:

- For class 0: precision = 1.00, recall = 0.97, and F1-score = 0.99 (36 samples);
- For class 1: precision = 0.99, recall = 1.00, and F1-score = 1.00 (125 samples).

Although the performance is better on average, if a more in-depth examination is carried out, it can be noted that there is only one misclassified sample, and hence it can be inferred that the Bagging model, unlike the decision tree model, does not completely avoid the possibility of misclassification.

#### 5.2.3. Performance of the Linear Discriminant Analysis Model

The Linear Discriminant Analysis (LDA) model achieved an overall accuracy of 0.8509; however, the detailed performance analysis reveals significant limitations for drinking water safety assessment. The confusion matrix for this model is:

$$\left[\begin{array}{cc} 7 & 22\\ 2 & 130 \end{array}\right]$$

- For class 0: precision = 0.78, recall = 0.24, and F1-score = 0.37 (29 samples);
- For class 1: precision = 0.86, recall = 0.98, and F1-score = 0.92 (132 samples).

The averages reported are based on a macro-averaged recall of 0.61 and a weighted average recall of 0.85. The low level of recall for the key class shows that a significant percentage of non-drinkable items are not correctly identified, resulting in a high rate of false negatives. Therefore, in spite of the relatively acceptable accuracy, the LDA model is not applicable for use in water safety.

#### 5.2.4. Performance of Neural Networks and Linear Regression

The Neural Network model has a test accuracy of 0.8696, while the loss value is 1.2310. Even though the accuracy of the model is high, the absence of competitive safety-related metrics affects the usability of the model in the assessment of drinking water safety. For Linear Regression, the results show that the model performs poorly, as the Mean Squared Error (MSE) is 0.13, while the coefficient of determination (R^2^) is 0.11, which shows that the model only manages to account for a small proportion of the variance in the data. This supports the claim that linear regression is an inappropriate model for binary classification, as in the case of drinking water potability assessments.

##### Summary of Results

The comparison indicates that there are various models that achieve good accuracy, while there is only one model that succeeds in eliminating false negatives. The model that succeeds in perfect achievements for precision, recall, and the F1-score is the decision tree model. The use of ensemble models, such as the Bagging Classifier, results in similar accuracy, although it does not guarantee that safety-critical samples will be detected. The limitations in the results provided by classical statistical models and neural networks in terms of safety-critical detection highlight the need for safety-oriented performance metrics in the evaluation of drinking water monitoring systems.

## 6. Conclusions

Having universal access to safe and clean drinking water remains an international priority in a context of increasing urbanization, aging mains, and various environmental demands. Our solution combines machine learning (ML), rule-based reasoning, and real-time cloud infrastructure to deliver a viable solution for the monitoring, categorization, and enhancement of water quality for the urban environment.

We applied twelve different ML algorithms over a dataset of 804 real-world water quality samples, each comprising 17 parameters from open-source AquaTim reports. Our results demonstrate the great potential that interpretable machine learning models may have as decision-support tools to be implemented in regulatory water quality systems. Particularly, the decision tree classifier highlighted a strong internal consistency of the evaluated dataset, reflecting how the compliance labels have a threshold-driven structure. The resulting high performance of the model highlights its ability to automate regulatory decision logic from real-world measurements. We also confirm previous results found in the literature, decision trees resulting in being the most suited for threshold-based classification problems such as water potability prediction. Although valuable for its exploratory, compliance-oriented decision-support framework, our proposed system also involves certain limitations that must be emphasized. The present study is based on a single municipal case study for Timișoara, Romania, and it relies upon a validation strategy of a single train–test split of the water quality data. The proposed system is, therefore, usable as a compliance-oriented decision-support framework capable of complementing regulatory procedures. Future work will diversify operational conditions and also integrate validation strategies with a higher degree of complexity.

In the present study, we have also addressed the absence of holistic systems capable of both predicting water quality levels and providing users with potential remedies. With the inclusion of WHO standards and rule-based decision logic, the system is able to detect anomalies and suggest simple but nonetheless meaningful treatment solutions, such as UV disinfection or activated carbon filtration. This is a unique trait compared to the previous literature, which is mostly concerned with prediction without going to the extent of post-diagnostic action or user-level output.

Another major strength is the practical field deployability and scalability of the system. The use of Google Firebase for storage enables the design of a real-time, updatable, and universally accessible database. Our system, unlike the majority of the studies related to it, which are conceptual or are feasible for use with only offline datasets, is capable of handling continuous ingestions of novel samples and real-time prediction. The database insertion includes spatial and temporal metadata, enabling the analysis for location-based studies and the observation of long-term trends. It is useful for the utility operator and the municipal planner to understand spatial inequities of water quality and to tailor the intervention accordingly for optimization.

Case studies from Timișoara yielded some practical conclusions that underscore the value of the system. The analysis highlighted that 19.8% of all water samples did not conform to the WHO standards of water potability, particularly the total organic carbon (TOC) and the microbiological contamination have been the two leading causes of the deviation. Some neighborhoods, for instance, Girocului and Complex Studențesc, showed higher occurrence rates for water quality deficiencies, whereas the Kiriac area always showed the lowest rate for conformity with the WHO standards. Such fine-grained intelligence can help city administrators use capital investments in infrastructure, determine the sources of contamination, and also design public health communication strategies.

Furthermore, the comparison among the ML models is also crucial for potential developers and researchers. Although the decision tree and gradient boosting classifier showed the best performance, other models, for example, SVM and KNN, showed poor performance for this binary classification task. Notably, the proposed system supports dynamic model selection for the ML models; thus, it is the right choice for researchers with differing performance, interpretability, and computation needs, other than decision-makers.

The system is also suitable for future extensions, allowing for potential additions of Internet of Things (IoT) sensors, robots for automatic data scraping, or even end-user-reported results from mobile clients. Such an extension can allow for a shift from reactive to proactive water management, with the detection and correction of exception cases before these become public health emergencies. Our system also utilizes open-source software and publicly accessible data, being easily adaptable for implementation within other cities or territories that publish regular water quality reports.

The simplicity of the system’s interface, together with the adherence to international norms, renders the system capable of providing access to actionable water quality information. The system enables informed actions at both the household and policymaking levels.

Hydraulic modeling tools could also be integrated in future works to specifically account for pressure zones, residence time, stagnation, and mixing phenomena within the water distribution network. We intend to combine data-driven approaches with hydraulic simulations, such as EPANET models, in order to further enhance the robustness of the already implemented water quality decision-support systems.

The limitations inherent to the current implementation consist of the fact that our system was built using periodic public reports, is subject to latency, and lacks high levels of granularity. Also, whereas basic treatment advice is provided, the website does not, at this time, incorporate detailed chemical simulations. These limitations represent potential starting points for future research, especially in the integration of sophisticated water chemistry simulations.

One limitation of data-driven approaches in the field of water quality assessment is the possibility of tautological learning, which may be caused by the fact that machine learning models are trained with input variables directly used in the construction of compliance-based labels. Therefore, models may only learn deterministic threshold rules. For this reason, rule-based methods still remain essential for compliance verification, providing deterministic assessments that are also aligned with regulatory standards. The machine learning models we propose are not meant to replace rule-based decision logic, but rather, they are utilized as complementary tools to support decision-making. Our integrated models are useful for scenarios when rule-based approaches may not be sufficient, such as managing measurement uncertainty and supporting scalable, spatially distributed monitoring scenarios.

Finally, we highlight the advantage of integrating ML algorithms, cloud computing, and rule-based reasoning for enhancing water safety in urban settlements. The system proposed performs water potability classification while also adding the value proposition through intervention recommendations and detection of at-risk areas. Its successful implementation with real municipal datasets and extensibility telegraphs the potential to serve as a template for smart water quality monitoring systems for cities anywhere. In this way, it adds to the technical literature around the application of ML to environmental monitoring and to the overall goal of sustainable development and protection of public health.

### Future Perspectives

In this research work, the parameter and model selection process was conducted systematically, taking into consideration factors specific to the drinking water domain, as well as the constraints dictated by international public health standards. Parameters like colony counts at 22 °C and 37 °C, the presence of *Escherichia coli* and enterococci, total organic carbon (TOC), pH, and nitrate levels are the ones commonly used by regulatory bodies and water operators to assess compliance with the WHO guidelines. These parameters have thus been retained in their raw form to maintain the traceability of the model’s decisions with respect to the normative thresholds.

It is also important to mention that there are parameters with well-established microbiological relationships. For example, there is a relationship between colony counts at 22 °C and 37 °C, as well as bacteriological indicators like the presence of *Escherichia coli* and enterococci. Under an extended methodological approach, the correlations could be explored using dimension reduction techniques like PCA to identify orthogonal components and to better understand the correlation patterns between the variables. The implementation of PCA would imply the loss of the physical and normative meaning of the variables, which is not desirable in the drinking water domain.

In addition, it is important to note that the accuracy of the model may be further increased by applying other data-driven methods such as hyperparameter tuning, stratified cross-validation, and automated feature selection. However, these methods were not explored in the present study, as the primary objective was to develop a working, reproducible, and easily interpretable system that can be directly applied to real data collected from a public water distribution network. Therefore, the application of advanced correlation analysis, further refinement of the model, and the integration of advanced data reduction techniques have all been identified as specific objectives for further research that will be explored in a separate study.

The selection of the “Decision Tree” model was not random but, rather, informed by the well-established characteristics of the approach for the application of binary classification problems based on decision rules and thresholds. The approach is well known for its ability to handle non-linear relationships, correlated data, and, more importantly, for the ability of the resulting decision rules to be easily interpreted. The above factors render the approach particularly suitable for applications of critical importance in the domain of public health. Furthermore, the approach and the application of the “Decision Tree” and “Ensemble” variants have been extensively applied in industrial-scale applications for the development of classification and decision-support tools by major technology corporations. The accuracy of the decision tree model was exceptional, as demonstrated in this study, thus validating the model’s suitability to this particular problem, as well as World Health Organization potability standards, and their alignment with tree-based decision rules. The above implies that the model is able to automatically mimic the evaluation approach traditionally applied by domain experts.

## Figures and Tables

**Table 1:** Results obtained using ML algorithms.

Algorithm	Accuracy	AUC	PRC Area	Calibration Quality
Linear regression	0.78	0.78	–	Poor
Logistic Regression	0.86	0.93	High	Underconfident
Naïve Bayes	0.83	0.99	High	Moderate
Decision Tree	1.00	1.00	Perfect	Perfect
Random Forest	0.98	0.96	High	Weak
SVM	0.81	0.94	High	Irregular
KNN	0.86	0.75	Medium	Poor
LDA	0.85	0.78	Medium	Spiky
Bagging Classifier	0.988	0.97	High	Good
AdaBoost	0.996	0.99	High	Slightly Overconfident
Gradient Boosting	0.996	0.99	High	Excellent
MLP (Keras)	–	0.50	0.89	Poor

**Table 2:** Water quality results for Timișoara Public Water Network.

Class Distribution	Drinkable Water Samples	Not Drinkable Water Samples
Percentage	80.2%	19.8%
Water Drinkability Prediction	The Best ML Algorithm	Other Possible Fits
	Decision Tree	Random Forest Gradient Boosting Bagging Classifier
Water Recommendation Results		
Water with highest pH	Airport Area	
Water with lowest pH	Telegrafului Area	
Optimal water	Kiriac Area	
Area that needs most treatment solutions	Girocului and Complex Studențesc Areas	

**Table 3:** Comparison of ML water quality prediction studies.

No.	Authors and Reference	Water Data	Params	ML Algos	Best ML Algorithm	Accuracy
1	N. Radhakrishnan, A.S. Pillai [21]	Rivers in India	4	3	Decision Tree	98.5%
2	R. Alnaqeb, F. Alrashdi, K. Alketbi, H. Ismail [23]	Online dataset	20	5	LightGBM	99.74%
3	M.I. Khoirul Haq et al. [24]	Online dataset	9	2	Decision Tree	97.23%
4	A.N. Hasan, K.M. Alhammadi [25]	Abu Dhabi	7	5	Decision Tree	97.70%
5	S. Kaddoura [26]	Online dataset	9	11	KNN and SVM	73.0%
6	E. Dritsas, M. Trigka [27]	Online dataset	20	10	Stacking Model	98.1%
7	Madni H.A. et al. [28]	Online dataset	9	7	Stacked Model	97.0%
8	**Current Study (Timișoara)**	Timișoara	17	12	**Decision Tree**	**100%**

## Data Availability

The raw data supporting the conclusions of this article will be made available by the authors on request.

## Abbreviations

The following abbreviations are used in this manuscript:

AI	Artificial Intelligence
ANN	Artificial Neural Network
API	Application Programming Interface
AUC	Area Under the Curve
DSS	Decision Support System
IoT	Internet of Things
JSON	JavaScript Object Notation
KNN	K-Nearest Neighbors
LDA	Linear Discriminant Analysis
LightGBM	Light Gradient Boosting Machine
LSTM	Long Short-Term Memory
MARS	Multivariate Adaptive Regression Splines
ML	Machine Learning
MLP	Multi Layer Perceptron
MSE	Mean Squared Error
PDF	Portable Document Format
pH	Potential of Hydrogen
ROC	Receiver Operating Characteristic
SVM	Support Vector Machine
TOC	Total Organic Carbon
WQI	Water Quality Index
WHO	World Health Organization
